# CircRNA‐mTOR Promotes Hepatocellular Carcinoma Progression and Lenvatinib Resistance Through the PSIP1/c‐Myc Axis

**DOI:** 10.1002/advs.202410591

**Published:** 2025-04-15

**Authors:** Yongchang Tang, Feng Yuan, Mingbo Cao, Yupeng Ren, Yuxuan Li, Gaoyuan Yang, Zhaozhong Zhong, Hao Liang, Zhiyong Xiong, Zhiwei He, Nan Lin, Meihai Deng, Zhicheng Yao

**Affiliations:** ^1^ Department of Hepatobiliary Surgery The Third Affiliated Hospital Sun Yat‐sen University Guangzhou 510630 China; ^2^ Department of General Surgery Qilu Hospital Shandong University Jinan 250012 China; ^3^ Department of Hepatobiliary Surgery The First Affiliated Hospital Guangzhou Medical University Guangzhou 510120 China; ^4^ Department of Kidney Transplantation The Third Affiliated Hospital Sun Yat‐sen University Guangzhou 510630 China; ^5^ Department of Hepatobiliary and Pancreatic Surgery The Third Affiliated Hospital Sun Yat‐sen University Guangzhou 510630 China

**Keywords:** c‐Myc, circRNA, hepatocellular carcinoma, Lenvatinib resistance, PSIP1

## Abstract

Circular RNAs (circRNAs) are crucial regulators of targeted drug resistance in hepatocellular carcinoma (HCC). However, the specific mechanisms underlying resistance that significantly hampers the effectiveness of HCC treatments remain unclear. Here, it is found that circRNA‐mTOR is highly expressed in HCC and strongly correlated with patient prognosis. Furthermore, circRNA‐mTOR enhances the stemness of HCC cells, thereby promoting the progression of HCC and contributing to lenvatinib resistance. Mechanistically, circRNA‐mTOR promotes the nuclear translocation of the RNA‐binding protein (RBP) PC4 and SRSF1 interacting protein 1 (PSIP1) through specific binding. The enhancement of HCC cell stemness by circRNA‐mTOR occurs via the PSIP1/c‐Myc signaling pathway, ultimately driving HCC progression and lenvatinib resistance. This study highlights the important role of circRNA‐mTOR in HCC progression and the maintenance of lenvatinib resistance and underscores its potential as a biomarker for the diagnosis and prognosis of HCC. In conclusion, this study provides an experimental foundation for targeted drug therapy in HCC and offers novel insights, perspectives, and methodologies for understanding the development and occurrence of this disease. These findings are significant for the development of new diagnostic and therapeutic markers for HCC, with the ultimate goal of reducing drug resistance.

## Graphical abstract

This study verifies that circRNA‐mTOR exhibits high expression levels in HCC and has a strong correlation with patient prognosis. Mechanistically, circRNA‐mTOR achieves this effect by facilitating the nuclear translocation of RNA‐binding protein (RBP) PSIP1 through specific binding. The promotion of HCC cell stemness by circRNA‐mTOR occurs via the PSIP1/c‐Myc signaling pathway, ultimately driving HCC progression and lenvatinib resistance.

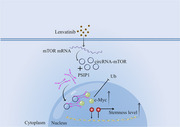

## 1 Introduction

Hepatocellular carcinoma (HCC) is a major malignant tumor that poses a significant threat to human health, with high morbidity and mortality rates.^[^ ^1^, ^2^ ^]^ Systemic therapy—particularly targeted therapy—plays a crucial role in the primary treatment approach for patients with advanced HCC who are ineligible for surgery or who experience extensive recurrence. The targeted drugs currently used in the systemic treatment of HCC are primarily multi‐targeted kinase inhibitors. Among these, lenvatinib has been shown to be non‐inferior to sorafenib in terms of overall survival (OS). Moreover, lenvatinib demonstrates significant improvements in all secondary endpoints, including higher objective response rate, longer time to progression, and progression‐free survival, compared with sorafenib.^[^ ^3^, ^4^ ^]^ While lenvatinib is effective in clinical practice, the development of acquired resistance due to drug resistance mutations is common under the selective pressure of molecular‐targeted therapy.^[^ ^5^ ^]^ Studies have shown a significant correlation between increased tumor stemness and lenvatinib resistance in HCC.^[^ ^6^ ^]^ However, the underlying mechanism of lenvatinib resistance, particularly the specific resistance targets in HCC, remains poorly understood. This gap in our understanding impacts the rational use of lenvatinib and clinical decision‐making for HCC treatment.

Circular RNAs (circRNAs) are single‐stranded molecules characterized by a covalently closed‐loop structure and tissue‐specific expression patterns during development.^[^ ^7^ ^]^ Due to their high abundance, stability, and unique expression patterns, circRNAs have been increasingly recognized for their significant regulatory roles in various biological processes, including tumor development and drug resistance. Importantly, several studies have reported the substantial impact of circRNAs on targeted drug resistance in HCC. E.g., one study found that circRNA‐SORE was highly expressed in sorafenib‐resistant HCC cells. CircRNA‐SORE binds to the major oncogenic protein YBX1 in the cytoplasm, thereby preventing the nuclear interaction between Y‐box binding protein 1 (YBX1) and the E3 ubiquitin ligase precursor mRNA processing factor 19 (PRP19). Consequently, the degradation of YBX1 mediated by PRP19 was inhibited, resulting in enhanced sorafenib resistance in HCC.^[^ ^8^ ^]^ In another study, researchers found that m6A modification promoted circRNA‐SORE expression, which acted as a microRNA (miRNA) sponge, separating miR‐103a‐2‐5p and miR‐660‐3p. This competitive activation of the Wnt/β‐catenin pathway induced sorafenib resistance.^[^ ^9^ ^]^ These findings emphasize the crucial regulatory role played by circRNAs in mediating targeted drug resistance in HCC; however, further research is needed.

In this study, we identified a highly expressed circRNA (circRNA_0 009792) in HCC, which was named circRNA‐mTOR because it originates from the mTOR gene. We noticed that circRNA‐mTOR significantly enhanced HCC tumor stemness and lenvatinib resistance. Mechanistic investigations revealed that circRNA‐mTOR interacts with PC4 and SRSF1 interacting protein 1 (PSIP1), leading to increased nuclear translocation of PSIP1, thus promoting tumor stemness enhancement.

## 2 Results

### 2.1 Screening and Identification of circRNA‐mTOR in HCC

**Figure 1:**
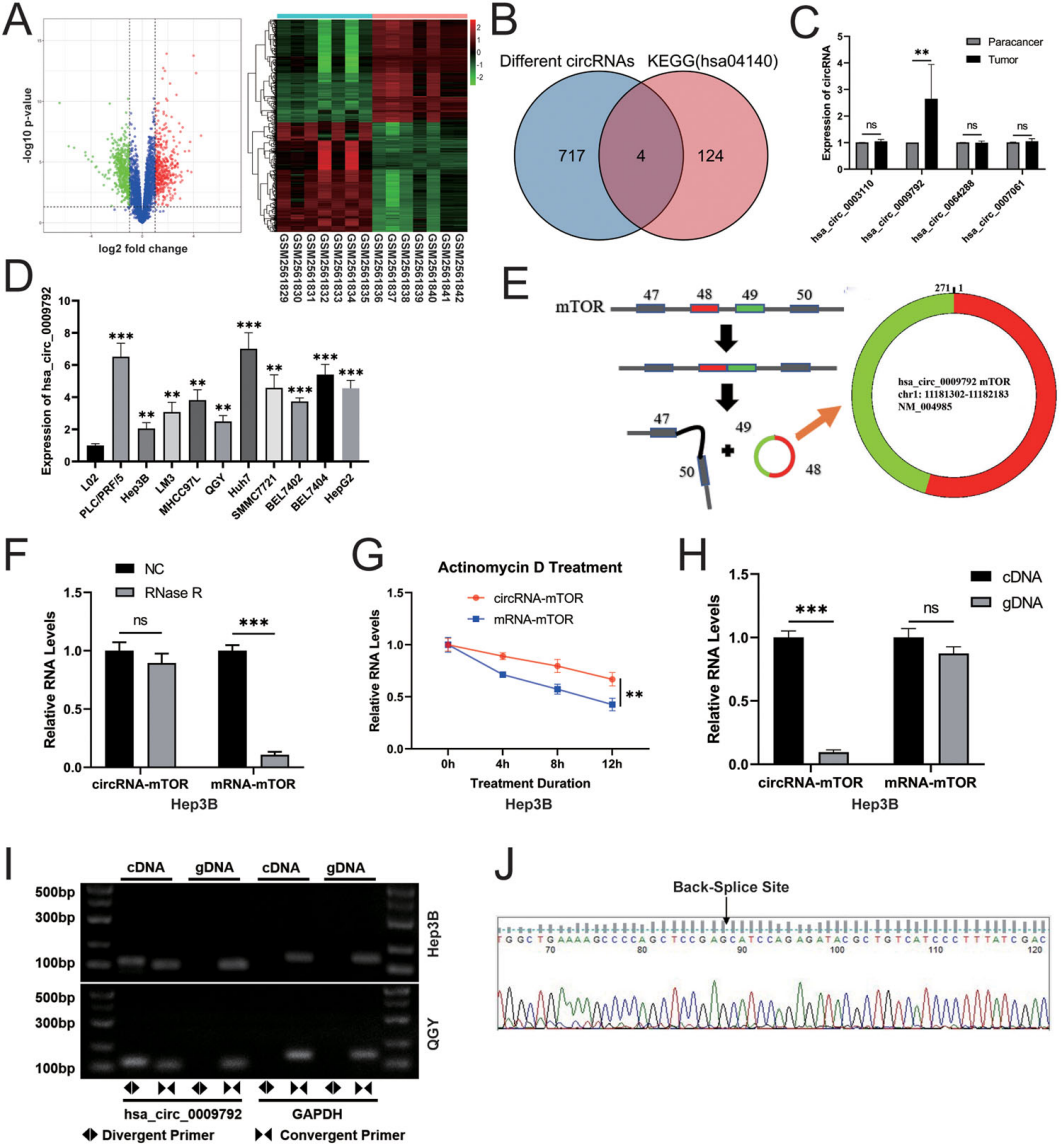
Screening and identification of circRNA‐mTOR in HCC. A) Volcano map and heat map of differentially expressed circRNAs; B) Venn diagram; C) The expression level of four up‐regulated circRNAs in eight pairs of HCC tissues and paired adjacent tissues, n = 8, paired *t*‐test; D) The expression level of hsa_circ_0 009792 in L02 and multiple HCC cell lines; E) The relevant information pattern diagram of hsa_circ_0 009792; F) RNase R resistance assay; G) Actinomycin D treatment experiment; H) Amplification of linear RNA (mRNA‐mTOR) and circRNA (circRNA‐mTOR) in gDNA and cDNA libraries; I) Agarose gel electrophoresis of PCR products; J) Sequencing analysis of PCR products. **p < 0.01; ***p < 0.001; ns: no significance.

The “Limma” package (REF) was used to perform differential expression analysis of circRNA‐related data from the Gene Expression Omnibus database (GSE97332). In total, 863 differentially expressed circRNAs were identified in HCC samples, including 417 upregulated and 446 downregulated circRNAs (|logFC| < 1) (**Figure** **1**A; File 1, Supporting Information). As autophagy may play a crucial role in the development of targeted drug resistance in HCC samples,^[^ ^10^ ^]^ we intersected the parent genes of these differentially expressed circRNAs with genes in the Kyoto Encyclopedia of Genes and Genomes database (hsa04140 dataset), resulting in four intersecting genes: AMBRA1, mTOR, ATG7, and PIK3C3 (Figure 1B; File 2, Supporting Information). These four genes encode the differentially expressed circRNAs hsa_circ_0 003110, hsa_circ_0 009792, hsa_circ_00 64288, hsa_circ_0 007061, and hsa_circ_0 005711 (**Table** **1**).

**Table 1:** Information on differentially expressed circRNAs identified through screening.

ID	logFC	t	*p* value	B	State	GeneSymbol
hsa_circ_0003110	1.370786	3.576844	0.003076	−2.58994	up	AMBRA1
hsa_circ_0009792	1.234187	4.505676	0.000505	−0.75952	up	MTOR
hsa_circ_0064288	1.079457	5.690546	5.78E‐05	1.468806	up	ATG7
hsa_circ_0007061	1.060137	4.407437	0.000609	−0.95089	up	AMBRA1
hsa_circ_0005711	−1.69812	−8.21204	1.08E‐06	5.598315	down	PIK3C3

Since our study primarily focused on enhancing the understanding of drug resistance mechanisms, we selected a key circRNA from the four most upregulated circRNAs according to previous research on HCC. To identify key circRNAs, we examined their expression using RT‐qPCR in eight pairs of HCC and paracancerous tissues. The results showed that the expression of hsa_circ_0 009792 in HCC tissues was significantly higher than that in paracancerous tissues (P = 0.0087). However, no significant differences were found in the expression levels of hsa_circ_0 003110 (P = 0.1513), hsa_circ_00 64288 (P = 0.6268), and hsa_circ_0 007061 (P = 0.2848) (Figure 1C). These findings indicated that hsa_circ_0 009792 was the key circRNA in our study. To further validate this result, we assessed the expression of hsa_circ_0 009792 in various HCC cell lines as well as in the normal liver cell line L02. The results demonstrated the upregulation of hsa_circ_0 009792 in the HCC cell lines compared to the expression in L02 cells (Figure 1D). Therefore, we identified hsa_circ_0 009792, also known as circRNA‐mTOR, as a key circRNA, which was used for subsequent validation experiments.

Using the circBase database and the University of California Santa Cruz databases, we determined that hsa_circ_0 009792 was derived from the 48th and 49th exons of the mTOR gene (Figure 1E). To further verify the role of hsa_circ_0 009792, we conducted an RNase R resistance experiment, which revealed that circRNA‐mTOR exhibited stronger resistance to RNase R compared to linear RNA (mRNA‐mTOR) (*P* < 0.0001) (Figure 1F). In addition, an actinomycin D experiment demonstrated that circRNA‐mTOR was more stable than mRNA‐mTOR (Figure 1G). We further detected the amplification of mRNA‐mTOR and circRNA‐mTOR in the gDNA and cDNA libraries, respectively. These results indicated that circRNA‐mTOR was only amplified in the cDNA library, whereas mRNA‐mTOR was significantly amplified in both the gDNA and cDNA libraries (Figure 1H). These findings were confirmed using agarose gel electrophoresis of the PCR products, which showed consistent results (Figure 1I). Moreover, sequencing analysis confirmed the consistency between the sequencing and the database findings and identified a back‐splice site (Figure 1J).

### 2.2 Clinical Relevance of circRNA‐mTOR in HCC

**Figure 2:**
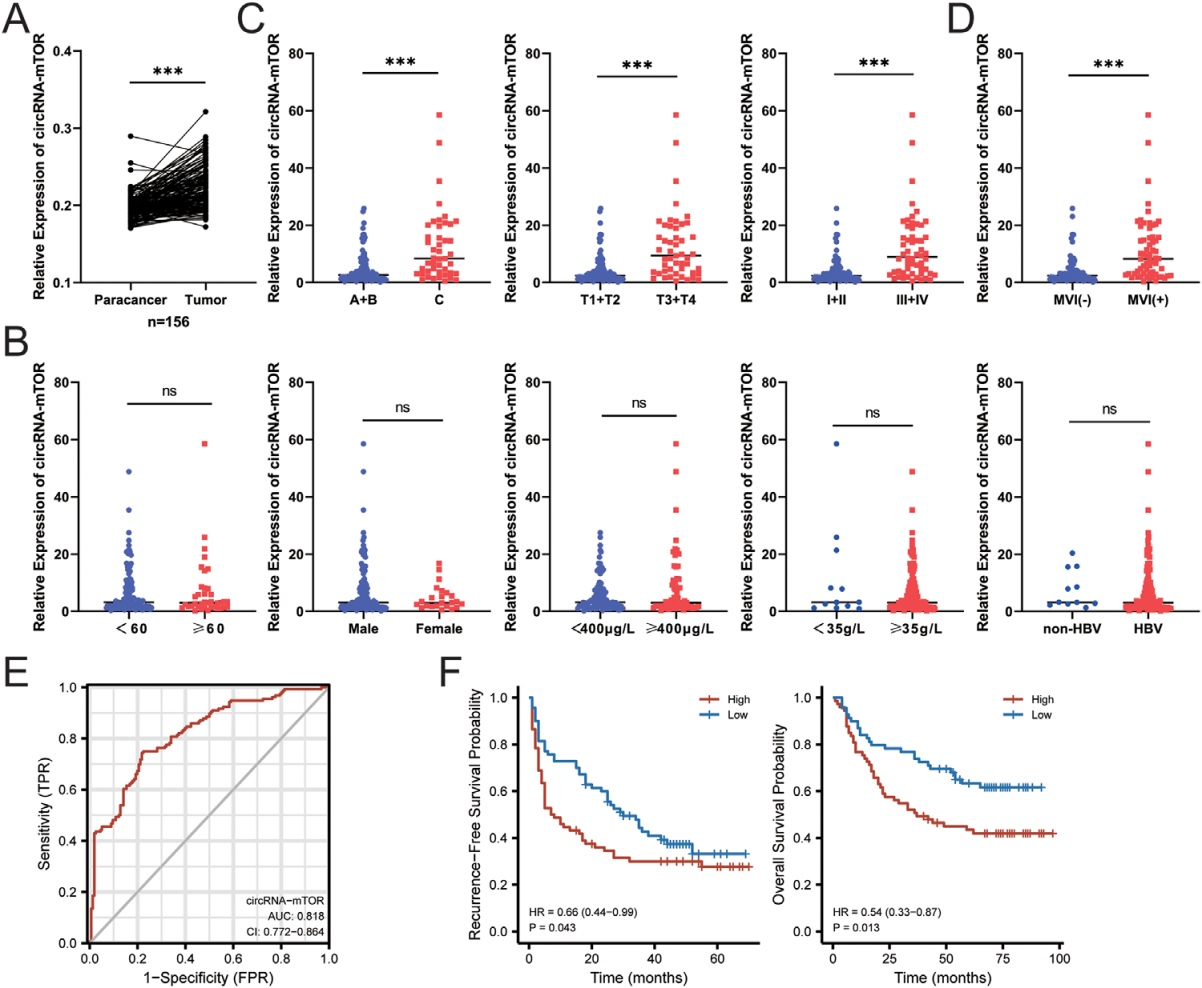
The clinical relevance of circRNA‐mTOR in HCC. A) The expression of circRNA‐mTOR in tissue samples, n = 156, paired *t*‐test; B) circRNA‐mTOR was not associated with age (left 1), gender (left 2), alpha‐fetoprotein level (middle), albumin level (right 2), or HBV infection (right 1), n = 156; C) circRNA‐mTOR was significantly correlated with BCLC stage (left), TNM‐T stage (middle), and tumor grade (right), n = 156; D) circRNA‐mTOR was significantly correlated with MVI, n = 156; E) ROC curve, n = 156; F) K‐M curve of the correlation between circRNA‐mTOR and RFS/OS, n = 156. ***p < 0.001; ns: no significance.

A total of 156 pairs of HCC and paracancerous tissues were used to investigate the association between circRNA‐mTOR and clinical indicators (**Table** **2**). Analysis of circRNA‐mTOR expression in tissues revealed significant overexpression of circRNA‐mTOR in HCC tissues compared to that in the paracancerous tissues (**Figure** **2**A). Based on these findings, we examined the relationships between circRNA‐mTOR and various clinical parameters. The results demonstrated that circRNA‐mTOR did not exhibit significant correlations with age (categorized as < 60‐ and ≥ 60‐years of age), gender (male and female), alpha‐fetoprotein level (categorized as <400 µg L⁻^1^ and ≥ 400 µg L⁻^1^), albumin level (categorized as < 35 g L⁻^1^ and ≥ 35 g L⁻^1^), or HBV infection (with and without) with P values of 0.888, 0.221, 0.092, 0.057, 0.746, respectively (Figure 2B and Table 2).

**Table 2:** Baseline data of clinical samples.

Characteristics		Counts (n = 156)	Relative expression levels of circRNA‐mTOR			*p* value
			High (n = 78)		Low (n = 78)	
Age (years)	< 60	118	61	57		0.888
	≥ 60	38	17	21		
Gender	male	133	71	62		0.221
	female	23	7	16		
Alpha‐fetoprotein (µg L⁻^1^)	< 400	92	48	44		0.092
	≥ 400	64	30	34		
Albumin (g L⁻^1^)	< 35	12	7	5		0.057
	≥ 35	144	71	73		
HBV infection	No	11	7	4		0.746
	Yes	145	71	74		
BCLC	A + B	111	46	65		< 0.001
	C	45	32	13		
TNM‐T	T1 + T2	107	43	64		< 0.001
	T3 + T4	49	35	14		
Grade	I + II	100	40	60		< 0.001
	III + IV	56	38	18		
MVI	negative	94	36	58		< 0.001
	positive	62	42	20		

However, a significant correlation was observed between circRNA‐mTOR and the BCLC stage (categorized as A + B and C) (*p* < 0.001), TNM‐T stage (categorized as T1+T2 and T3+T4) (*p* < 0.001), and grade (categorized as I + II and III + IV) (*p* < 0.001). Specifically, the expression levels of circRNA‐mTOR were higher in patients with advanced BCLC stage, TNM‐T stage, and higher tumor grade (Figure 2C). Additionally, a significant correlation was found between circRNA‐mTOR and microvascular invasion (MVI) (categorized as MVI(+) and MVI(‐); *p* < 0.001). The expression level of circRNA‐mTOR was elevated in MVI(+) cells (Figure 2D).

Next, receiver operating characteristic curve analysis was conducted, which revealed an area under the curve value of 0.818 (95% confidence interval (CI): 0.772‐0.864) for circRNA‐mTOR, indicating good diagnostic power (Figure 2E). Subsequently, the circRNA‐mTOR expression in HCC tissues was divided into high‐ and low‐expression groups using the median as the cutoff value. The correlation between circRNA‐mTOR and the recurrence‐free survival (RFS) and OS of patients was analyzed. The results of the Kaplan‐Meier (K‐M) survival curve analysis demonstrated a significant correlation between circRNA‐mTOR expression levels and the RFS and OS (*p* = 0.043 and 0.013, respectively). This indicated that patients with high circRNA‐mTOR expression had worse RFS and OS outcomes (Figure 2F).

### 2.3 CircRNA‐mTOR Promotes HCC Progression

**Figure 3:**
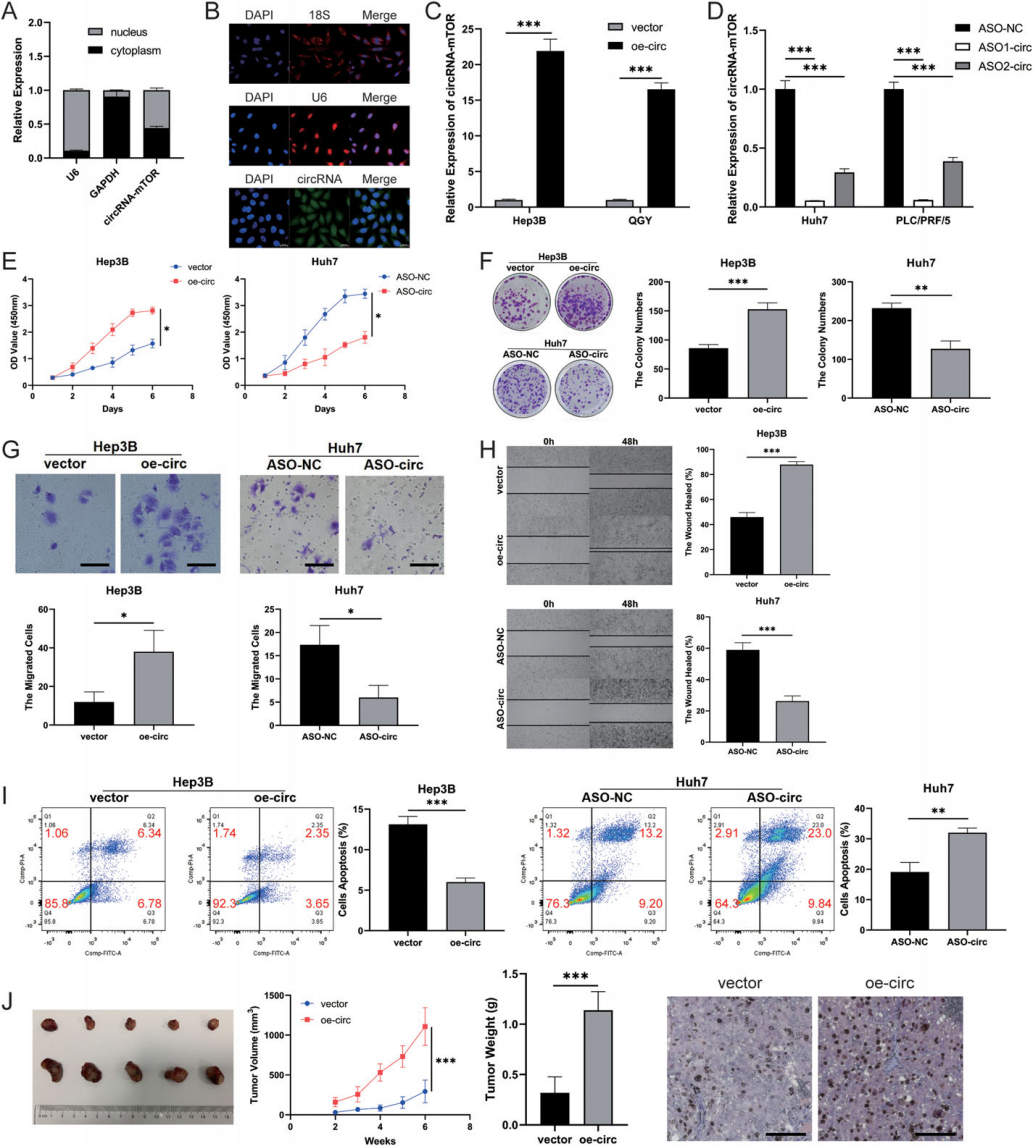
CircRNA‐mTOR promotes HCC progression. A) CircRNA‐mTOR was localized in the nucleus and cytoplasm, Scale bar, 50 µm; B) FISH assay showed the cellular localization of circRNA‐mTOR; C) The construction of Hep3B and QGY cell lines overexpressing circRNA‐mTOR; D) The construction of Huh7 and PLC/PRF/5 cell lines interfering with circRNA‐mTOR; E) CCK8 cell proliferation assay; F) Clone formation assay; G) Transwell invasion assay, Scale bar, 50 µm; H) Wound Healing assay; I) Flow cytometry was used to detect apoptosis; J) Subcutaneous tumor formation of nude mice in the vector group and overexpression circRNA‐mTOR (oe‐circ) group; Physical image of the tumor (left); Comparison of tumor growth curve and weight between the two groups (middle); Immunohistochemical staining results of Ki67 expression levels of tumors in the two groups (right), Scale bar, 50 µm. *p < 0.05; **p < 0.01; ***p < 0.001.

To investigate the impact of circRNA‐mTOR in HCC, we examined its expression levels in the nucleus and cytoplasm using an RNA nucleoplasmic separation assay and RT‐qPCR. The findings revealed that circRNA‐mTOR was present in both the nucleus (55.8%) and cytoplasm (44.2%) relative to the nuclear reference U6 and the cytoplasmic reference GAPDH (**Figure** **3**A). Additionally, we conducted fluorescence in situ hybridization (FISH) experiments, which demonstrated that circRNA‐mTOR, labeled with green fluorescence, was detected in both the nucleus and cytoplasm compared to the nuclear reference U6 and the cytoplasmic reference 18S (Figure 3B).

To further investigate the function and mechanism of circRNA‐mTOR, we constructed an overexpression vector for circRNA‐mTOR and successfully established Hep3B and QGY cell lines overexpressing it (Figure 3C). Moreover, we created two ASO expression interference systems in Huh7 and PLC/PRF/5 cell lines (Figure 3D). Subsequently, several assays were performed to assess the effects of circRNA‐mTOR. The CCK8 cell proliferation assay demonstrated that circRNA‐mTOR promoted HCC cell proliferation in vitro (Figure 3E; Figure S1A, Supporting Information). The clone formation assay revealed that circRNA‐mTOR enhanced the ability of HCC cells to proliferate indefinitely in vitro (Figure 3F; Figure S1B, Supporting Information). Additionally, the Transwell invasion assay indicated that circRNA‐mTOR augmented the invasiveness of HCC cells in vitro (Figure 3G; Figure S1C, Supporting Information). Moreover, the wound healing assay demonstrated that circRNA‐mTOR facilitated the migration of HCC cells in vitro (Figure 3H; Figure S1D, Supporting Information). Furthermore, a flow cytometry apoptosis detection assay showed that circRNA‐mTOR reduced apoptosis rates in HCC cells in vitro (Figure 3I; Figure S1E, Supporting Information).

Subsequently, we conducted in vivo experiments using animal models, which revealed that circRNA‐mTOR increased the volume and weight of tumors, and also their Ki67 expression level compared to those in the control group (Figure 3J; Figure S1F, Supporting Information). Collectively, our experimental findings strongly indicate that circRNA‐mTOR promotes HCC progression.

### 2.4 CircRNA‐mTOR Promotes Lenvatinib Resistance and Improvement of Tumor Stemness in HCC

**Figure 4:**
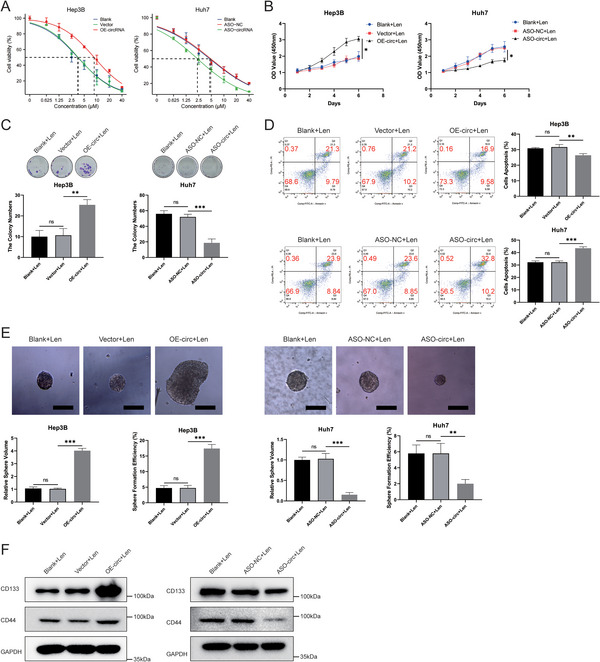
CircRNA‐mTOR promotes lenvatinib resistance and the improvement of tumor stemness in HCC. A) CircRNA‐mTOR significantly increased the lenvatinib IC50 value of HCC cells. The IC50 value for Hep3B: blank 3.512 (3.342‐3.681) µm, vector 3.586 (3.417‐3.754) µm, OE‐circRNA 8.656 (8.246‐9.065) µm; The IC50 value for HuH7: blank 4.557 (4.34‐4.773) µm, ASO‐NC 4.836 (4.609‐5.064) µm, ASO‐circRNA 2.327 (2.219‐2.436) Μm. B) CircRNA‐mTOR promoted the proliferation ability of HCC cells when lenvatinib was treated; C) CircRNA‐mTOR promoted the colony formation ability of HCC cells when lenvatinib was treated; D) CircRNA‐mTOR down‐regulated the apoptosis level of HCC cells when lenvatinib was treated; E) CircRNA‐mTOR promoted the stemness level of HCC cells when lenvatinib was treated by sphere formation experiment, Scale bar, 100 µm; F) CircRNA‐mTOR promoted the increase in protein levels of HCC related stemness markers (CD133, CD44). *p < 0.05; **p < 0.01; ***p < 0.001.

To investigate the effect of circRNA‐mTOR on lenvatinib resistance in HCC, we initially evaluated the changes in the IC50 value of lenvatinib. Our results showed that HCC cell lines exhibited an increased lenvatinib IC50 value after overexpressing circRNA‐mTOR, whereas interfering with circRNA‐mTOR expression led to a decreased lenvatinib IC50 value in HCC cell lines (**Figure** **4**A; Figure S2A, Supporting Information).

Subsequently, we treated HCC cells with lenvatinib and conducted cell function experiments to explore its effects of circRNA‐mTOR on these cells. The findings revealed that circRNA‐mTOR promoted the proliferation and clone formation of lenvatinib‐treated HCC cells while reducing their apoptosis rates compared to those of the control group (Figure 4B–D; Figure S2B–D, Supporting Information).

To investigate whether circRNA‐mTOR enhances lenvatinib resistance in HCC by altering tumor stemness, we conducted a sphere‐formation experiment. The results indicated that in the presence of lenvatinib, overexpression of circRNA‐mTOR significantly enhanced the sphere‐forming ability of HCC cells, whereas curtailing circRNA‐mTOR expression significantly reduced the sphere‐forming ability of HCC cells (Figure 4E; Figure S2E, Supporting Information). At the same time, we detected HCC related tumor stemness markers (CD44 and CD133) in these spheres and found that circRNA‐mTOR significantly promoted the expression of stemness markers (Figure 4F). Overall, our findings strongly suggest that circRNA‐mTOR promotes lenvatinib resistance in HCC and contributes to enhanced tumor stemness.

### 2.5 CircRNA‐mTOR Promotes Progression and Lenvatinib Resistance of HCC by Binding to PSIP1 Causing Its Nuclear Translocation

The mechanism of action of circRNA‐mTOR was also explored. First, the effect of circRNA‐mTOR on mRNA and protein levels of its parent gene, mTOR, were investigated. These results demonstrated that circRNA‐mTOR does not influence mTOR mRNA expression in HCC cells (Figure S3A, Supporting Information). Additionally, circRNA‐mTOR does not affect the protein expression of mTOR nor that of other key proteins involved in the autophagy pathway, such as p62 and LC3B (Figure S3B, Supporting Information). These findings suggest that circRNA‐mTOR does not affect the expression of its parent gene nor the modulation of autophagy in HCC.

**Figure 5:**
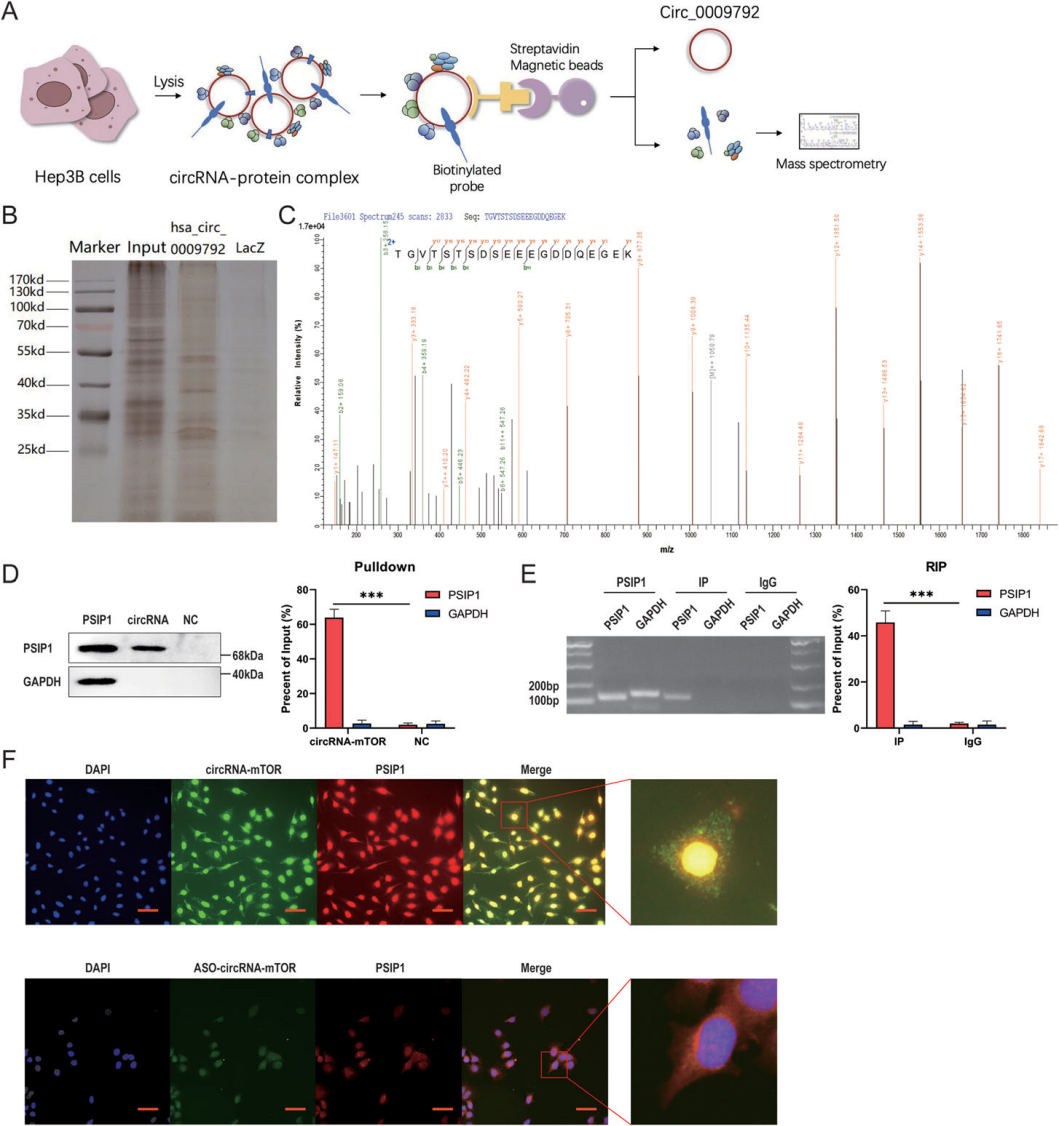
RNA binding protein PSIP1 is a key protein in the downstream mechanism of circRNA‐mTOR, and they can specifically bind to each other. A) Schematic diagram of RNA Pulldown experiment and LC‐MS experiment; B) Result of protein silver stain after Pulldown experiment; C) Peak mass spectrum of PSIP1; D) Protein expression of PSIP1 after RNA Pulldown experiment; E) The result of circRNA‐mTOR been pulled down after RIP experiment; F) FISH and immunofluorescence co‐localization experiment result of circRNA‐mTOR and PSIP1, Scale bar, 50 µm. ***p < 0.001.

Considering that the interaction between circRNAs and RNA‐binding proteins (RBPs) is crucial for regulating tumor progression in HCC,^[^ ^11^ ^]^ this study focused on identifying downstream RBPs. RNA pull‐down and liquid chromatography–mass spectrometry (LC‐MS) experiments were conducted to identify the proteins that interacted with circRNA‐mTOR (**Figure** **5**A,B, **Table** **3**). Gene Ontology analysis and protein classification analyses were performed on the identified proteins (Figure S3C–F, Supporting Information). Based on the enhancement of tumor stemness and the results of LC‐MS and functional analyses, 16 RBPs with transcriptional regulatory activity were selected from the differentially expressed proteins through bioinformatics analysis, literature review, and analysis of RBP functions (**Table** **4**). Among these, PSIP1 had the highest protein score (Figure 5C, Table 4), making it a key RBP for further investigation.

**Table 3:** Differential proteins after pulldown experiment by circRNA‐mTOR (top 15 score).

Protein name	Protein accession	Protein score
TUBB4A	sp|P04350|TBB4A_HUMAN	356
ATAD3A	sp|Q9NVI7|ATD3A_HUMAN	269
TUBB6	sp|Q9BUF5|TBB6_HUMAN	222
MFN2	sp|O95140|MFN2_HUMAN	188
NELFE	sp|P18615|NELFE_HUMAN	180
ACSL4	sp|O60488|ACSL4_HUMAN	153
MCM2	sp|P49736|MCM2_HUMAN	139
LACTB	sp|P83111|LACTB_HUMAN	136
HSPA6	sp|P17066|HSP76_HUMAN	135
KRT19	sp|P08727|K1C19_HUMAN	132
LMO7	sp|Q8WWI1|LMO7_HUMAN	130
PSIP1	sp|O75475|PSIP1_HUMAN	127
MRPS27	sp|Q92552|RT27_HUMAN	126
IDH2	sp|P48735|IDHP_HUMAN	125
NAP1L4	sp|Q99733|NP1L4_HUMAN	114

**Table 4:** RBPs with transcriptional regulatory activity in the result of mass spectrometry analysis.

Protein name	Protein accession	Protein score
PSIP1	sp|O75475|PSIP1_HUMAN	127
SMARCA4	sp|P51532|SMCA4_HUMAN	79
KLF16	sp|Q9BXK1|KLF16_HUMAN	77
HDAC1	sp|Q13547|HDAC1_HUMAN	68
CDC5L	sp|Q99459|CDC5L_HUMAN	67
NME1	sp|P15531|NDKA_HUMAN	65
PIH1D1	sp|Q9NWS0|PIHD1_HUMAN	60
SMARCB1	sp|Q12824|SNF5_HUMAN	47
MTA2	sp|O94776|MTA2_HUMAN	44
ZNF787	sp|Q6DD87|ZN787_HUMAN	43
CREBBP	sp|Q92793|CBP_HUMAN	37
TCERG1	sp|O14776|TCRG1_HUMAN	34
HIC2	sp|Q96JB3|HIC2_HUMAN	33
SUB1	sp|P53999|TCP4_HUMAN	29
MED4	sp|Q9NPJ6|MED4_HUMAN	25
TSHZ3	sp|Q63HK5|TSH3_HUMAN	22

Subsequently, we validated the specific binding of circRNA‐mTOR to PSIP1. The results of the RNA pull‐down assay indicated that circRNA‐mTOR specifically interacted with PSIP1 compared to the control protein GAPDH (Figure 5D). Similarly, the RIP assay demonstrated specific binding of PSIP1 to circRNA‐mTOR compared to the IgG control (Figure 5E). Furthermore, FISH and immunofluorescence colocalization experiments revealed the colocalization of circRNA‐mTOR (green fluorescence) and PSIP1 (red fluorescence) in HCC cells (Figure 5F).

To investigate the role of PSIP1 in HCC progression and lenvatinib resistance, the expression and clinical significance of PSIP1 were analyzed using the Gene Expression Profiling Interactive Analysis (GEPIA) database to investigate its role in HCC progression and lenvatinib resistance. The results indicated that PSIP1 was highly expressed in HCC and that high expression of PSIP1 was associated with poor disease‐free survival and OS (*p* = 0.006 and 0.03, respectively (Figure S4A, Supporting Information). Subsequently, an interference system targeting PSIP1 was constructed, and its efficiency was verified (Figure S4B, Supporting Information). Functional experiments demonstrated that PSIP1 promoted the proliferation, colony formation, invasion, and metastasis of HCC cells as well as increased stemness and lenvatinib resistance (Figure S4C–H, Supporting Information).

**Figure 6:**
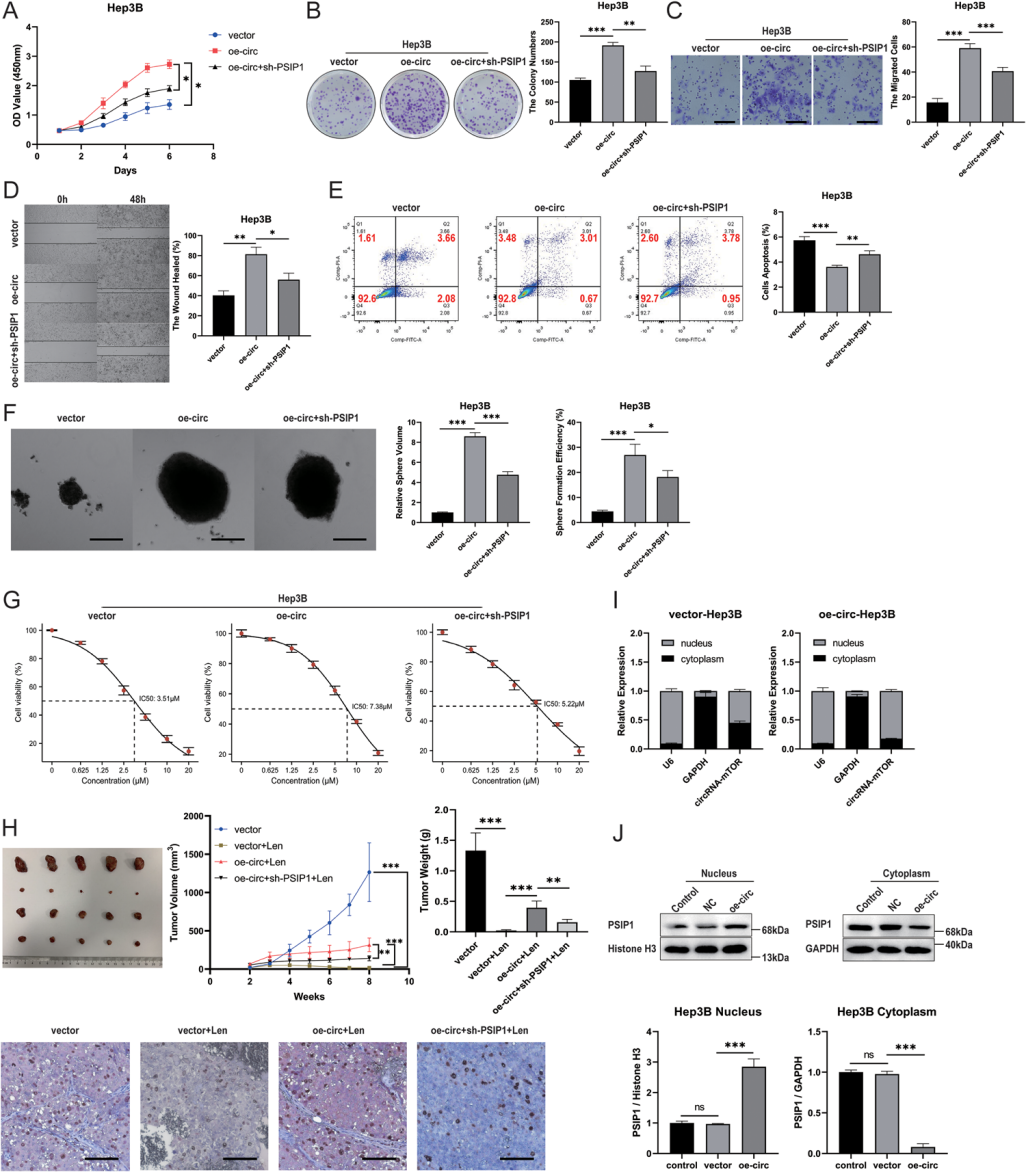
CircRNA‐mTOR promotes the progression of HCC and lenvatinib resistance by binding to PSIP1 and affecting its nuclear translocation. A–G) After interfering with PSIP1 expression, the promotion and improvement of circRNA‐mTOR on the proliferation ability, colony formation ability, invasion and metastasis ability, tumor stemness and IC50 value of lenvatinib of HCC cells could be partially reversed, and the inhibition of circRNA‐mTOR on the apoptosis of HCC cells could be partially reversed; A) CCK8 cell proliferation assay; B) Clone formation assay; C) Transwell invasion assay, Scale bar, 50 µm; D) Wound Healing assay; E) Flow cytometry was used to detect apoptosis; F) Sphere formation experiment, Scale bar, 100 µm; G) Lenvatinib IC50; H) Rescue experiments for animal models in vivo; Physical image of the tumor (above‐left); Comparison of tumor growth curve (above‐middle) and weight (above‐right) between the groups; Immunohistochemical staining results of Ki67 expression levels of tumors in the groups (below), Scale bar, 50 µm; I) The proportion of circRNA‐mTOR in the nucleus increased after overexpression; **J)** The proportion of PSIP1 in the nucleus increased after the overexpression of circRNA‐mTOR. *p < 0.05; **p < 0.01; ***p < 0.001; ns: no significance.

To further investigate whether circRNA‐mTOR affects HCC progression and lenvatinib resistance by interacting with PSIP1, rescue experiments were conducted by interfering with PSIP1 expression in HCC cells that stably overexpressed circRNA‐mTOR. The results showed that interference with PSIP1 expression partially reversed the promotional and enhancement effects of circRNA‐mTOR on proliferation, colony formation, invasion, metastasis, tumor stemness, and lenvatinib IC50 value in HCC cells and partially reversed the inhibitory effect of circRNA‐mTOR on apoptosis (**Figure** **6**A–G; Figure S5A–S5G, Supporting Information). Moreover, in vivo experiments using animal models demonstrated that interference with PSIP1 expression partially reversed circRNA‐mTOR's promotion of lenvatinib resistance, as evidenced by differences in tumor size, weight, and Ki67 expression (Figure 6H). These findings confirm that circRNA‐mTOR promotes HCC progression and lenvatinib resistance by binding to PSIP1.

Furthermore, the overexpression or knockdown of circRNA‐mTOR did not affect PSIP1 total protein expression (Figure S5H, Supporting Information). The results of RNA nucleoplasmic separation experiments showed that the proportion of circRNA‐mTOR was significantly higher in the nucleus of stably overexpressing cells compared to common HCC cells (Figure 6I; Figure S5I, Supporting Information). The results of protein nucleoplasmic separation experiments indicated that after overexpression of circRNA‐mTOR, the proportion of PSIP1 protein in the nucleus increased, while the proportion of PSIP1 protein in the cytoplasm decreased (Figure 6J; Figure S5J, Supporting Information). These results demonstrate that circRNA‐mTOR affects the nuclear translocation of PSIP1 by binding to it, thereby promoting the progression of HCC and lenvatinib resistance.

### 2.6 CircRNA‐mTOR Increases the Stemness Level of HCC Cells through PSIP1/c‐Myc Signaling Pathway

**Figure 7:**
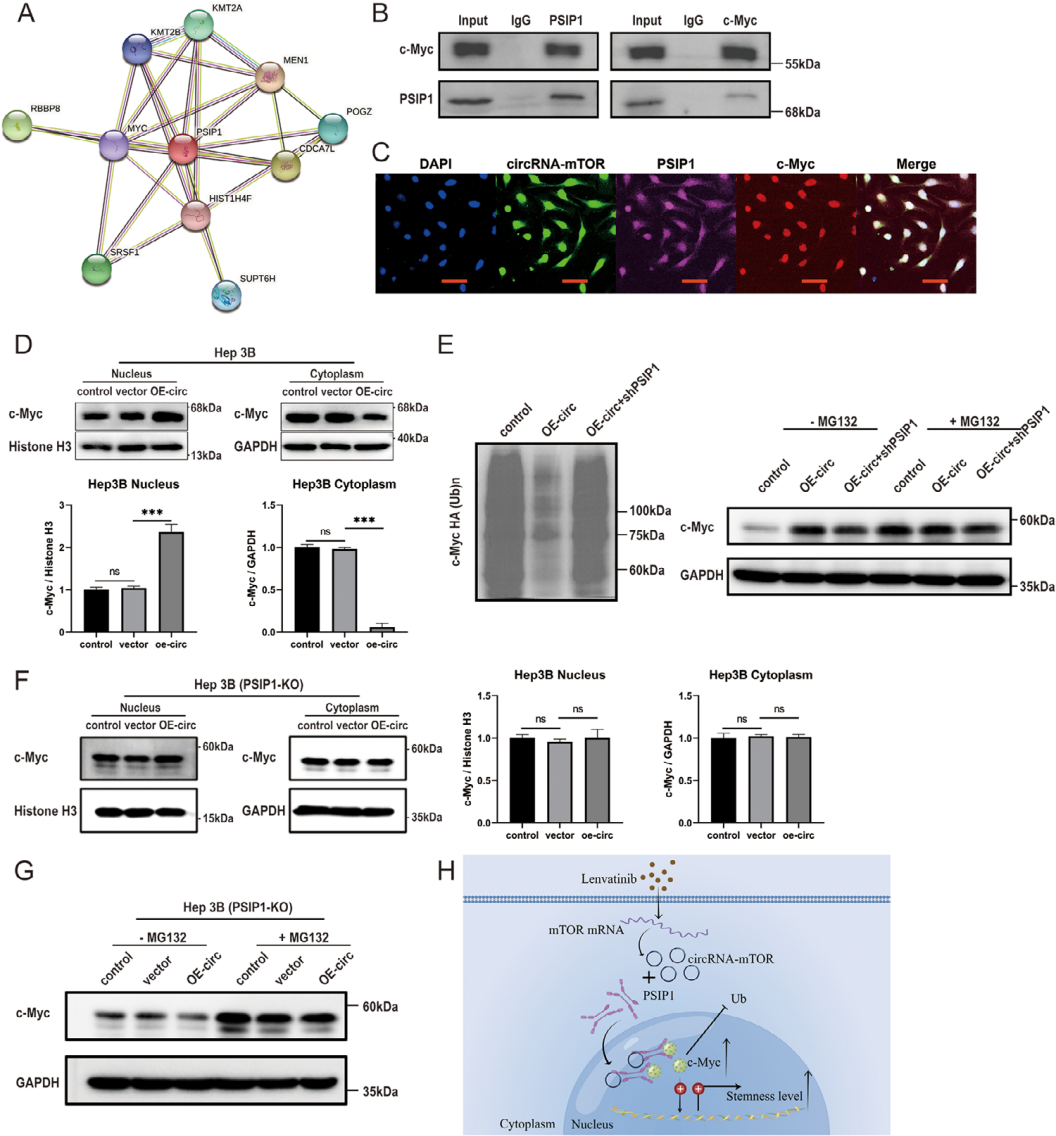
CircRNA‐mTOR increases the stemness level of HCC cells through PSIP1/c‐Myc signaling pathway. A) Proteins that interact with PSIP1 in the STRING database; MYC (c‐Myc) and PSIP1 could bind and were most closely connected; B) CO‐IP experimental results of the interaction between c‐Myc and PSIP1; C) FISH and immunofluorescence co‐localization of circRNA‐mTOR, PSIP1 and c‐Myc, Scale bar, 50 µm; D) The proportion of c‐Myc in the nucleus increased after the overexpression of circRNA‐mTOR; E) c‐Myc ubiquitination level detection; F) CircRNA‐mTOR cannot alter the localization of c‐Myc in PSIP1‐KO Hep3B cells; G) CircRNA‐mTOR cannot alter the stability of c‐Myc in PSIP1‐KO Hep3B cells. H) Mechanistic diagram of circRNA‐mTOR in HCC. Ub: ubiquitination.

To further investigate the downstream pathway of circRNA‐mTOR/PSIP1, we analyzed proteins that interacted with PSIP1 using the STRING database (https://cn.string‐db.org/). The results revealed that c‐Myc (MYC) and PSIP1 interact and are closely connected (**Figure** **7**A). This finding was further confirmed by a co‐immunoprecipitation (CO‐IP) experiment, which demonstrated specific binding between c‐Myc and PSIP1 (Figure 7B). Additionally, FISH and immunofluorescence colocalization experiments showed that circRNA‐mTOR, PSIP1, and c‐Myc exhibited common cellular localization in HCC cells (Figure 7C). Further research indicated that circRNA‐mTOR leads to increased retention of c‐Myc in the nucleus (Figure 7D). We speculated that due to the interaction between PSIP1 and c‐Myc, circRNA‐mTOR caused increased intranuclear transfer of PSIP1, leading to a decrease in c‐Myc extranuclear migration. The results of the ubiquitination detection and MG132 treatment experiments indicated that circRNA‐mTOR reduced the ubiquitination‐related degradation of c‐Myc, leading to an increase in c‐Myc levels in the nucleus, which could be partially reversed by PSIP1 (Figure 7E). To further confirm that the interaction between circRNA‐mTOR and c‐Myc is dependent on PSIP1, rather than a direct interaction between circRNA‐mTOR and c‐Myc, we constructed the PSIP1‐KO Hep3B cell line using CRISPR technology (Figure S5K, Supporting Information). The experimental results showed that the effect of circRNA‐mTOR on the nuclear localization and stability of c‐Myc was significantly reduced after PSIP1 knockout (Figure 7F,G). This further indicated the importance of PSIP1 in the circRNA‐mTOR/PSIP1/c‐Myc signaling pathway. Since c‐Myc is a recognized tumor stemness regulator, our experimental results suggested that circRNA‐mTOR enhances the stemness of HCC cells through the PSIP1/c‐Myc signaling pathway, thereby exerting its biological functions.

## 3 Discussion

Recent studies have identified increased stemness in HCC cells as a significant factor contributing to drug resistance.^[^ ^6^ ^]^ Importantly, in this study, we noticed a strong association between circRNA‐mTOR, HCC progression, and targeted drug‐resistance phenotypes. This association involved circRNA‐mTOR binding to PSIP1, an RBP that modulates its nuclear translocation, enhancing stemness in HCC cells. These findings provide novel insights into the mechanisms governing drug resistance in HCC (Figure 7H). Understanding the mechanisms underlying drug resistance and stemness regulation in HCC is crucial for developing effective therapeutic strategies. Targeting the circRNA‐mTOR/PSIP1 pathway could potentially overcome drug resistance and improve HCC treatment outcomes. Further research and validation are needed to translate these findings into clinical applications.

While previous transcriptomic studies have primarily focused on linear transcripts, circRNAs have gained recognition as important regulators of various biological processes, including tumor development. Due to their stability, high abundance, conservation, and tissue specificity, circRNAs are increasingly recognized for their biological functions.^[^ ^12^, ^13^ ^]^ Many studies have reported the impact of circRNAs on targeted drug resistance in HCC.^[^ ^8^, ^9^, ^14^, ^15^, ^16^ ^]^ Our study aimed to explore the effect and mechanism of circRNAs in HCC progression and drug resistance. We found that circRNA‐mTOR significantly enhanced the progression and stemness of HCC cells. Increased tumor stemness is known to be a key factor in drug resistance in HCC.^[^ ^6^ ^]^ Cancer stem cells (CSCs), a subset of stem/progenitor cells, drive changes in tissue hierarchy and contribute to tumor heterogeneity.^[^ ^17^ ^]^ Unlike typical tumor cells, CSCs possess a high capacity for self‐renewal, differentiation, and tumor formation, which contributes to their resistance to conventional therapies and increases the risk of tumor recurrence.^[^ ^18^ ^]^ The maintenance and regeneration of tumor stem cells depend on the activation of highly conserved stem cell signaling pathways.^[^ ^19^ ^]^ Importantly, the dysregulated activation of these pathways can lead to uncontrolled cell proliferation, aberrant differentiation, and tumorigenesis. Reactivation of these pathways can also prompt tumor reprogramming and the emergence of CSC phenotypes, which are involved in the onset and recurrence of HCC. Despite substantial research on HCC‐related CSCs, understanding the mechanisms that regulate their self‐renewal remains challenging.^[^ ^20^ ^]^ Numerous recent studies have highlighted the close association between circRNAs, other small molecular RNAs, and tumor stemness of HCC. E.g., Gu et al. identified high expression of a circular RNA named CircIPO11 in HCC tumor tissues and liver CSCs, establishing its role in maintaining liver CSC self‐renewal and initiating HCC development. This study also noticed that CircIPO11 recruited topoisomerase 1 (TOP1) to the glioma‐associated oncogene homolog 1 (GLI1) promoter, activating the Hedgehog signaling pathway.^[^ ^21^ ^]^ Another study demonstrated that deletion of circZKSCAN1 confers various malignant properties, including cancer stemness, and is linked to overall and RFS in HCC.^[^ ^22^ ^]^ Additionally, hsa_circ_00 67531 enhanced the pellet formation ability of HCC cells through CD90, promoting HCC tumor stemness.^[^ ^23^ ^]^ In agreement with the role of circRNAs in increasing stemness of HCC cells in these studies, our current study also demonstrates that circRNA‐mTOR promotes lenvatinib resistance by elevating the stemness of HCC cells.

Some studies have suggested that circRNAs influence their parent genes or autophagy in HCC.^[^ ^24^, ^25^ ^]^ Interestingly, our study did not notice any obvious effect of circRNA‐mTOR on mTOR or autophagy pathway, which required us to also study the underlying mechanism. In agreement with previous studies, we found that circRNA can function at multiple levels: (1) acting as miRNA inhibitors to regulate gene expression post‐transcriptionally (the “sponge” effect)^[^ ^7^, ^26^, ^27^ ^]^; (2) regulating gene transcription and RNA splicing^[^ ^28^, ^29^ ^]^; (3) serving as protein baits to regulate protein function^[^ ^30^ ^]^; (4) encoding functional peptide;^[^ ^31^ ^]^ (5) performing other confirmed functions.^[^ ^32^, ^33^, ^34^ ^]^ Based on our analysis, we propose that circRNA‐mTOR may function as a protein decoy to modulate protein function or mediate transcription regulation or differential cleavage. Numerous studies have demonstrated the significance of interactions between RBPs and circRNAs in tumor progression and HCC.^[^ ^11^ ^]^ For instance, circRHOT1 recruits TIP60 to the nuclear receptor subfamily 2 group F member 6 (NR2F6) promoter, initiating NR2F6 transcription and promoting HCC progression.^[^ ^35^ ^]^ Similarly, CircETFA promotes HCC progression by recruiting an RBP named EIF4A3 to prolong the half‐life of CCL5 mRNA.^[^ ^36^ ^]^ ESR1‐activated circ_0 004018 inhibits HCC angiogenesis by binding to a RBP named fused in sarcoma (FUS) and stabilizing TIMP2 expression.^[^ ^37^ ^]^ Another RBP named RBM3 promotes HCC cell proliferation by interacting with circular RNA SCD‐circRNA 2.^[^ ^38^ ^]^ CircCPSF6 competes with PCBP2 for binding to YAP1 mRNA, maintaining YAP1 stability and promoting the malignant progression of HCC.^[^ ^39^ ^]^ Consistent with these observations, our study identified PSIP1 as a key RBP that interacts with circRNA‐mTOR.

PSIP1, a transcriptional coactivator, has been linked to tumor stemness and drug resistance.^[^ ^40^, ^41^, ^42^, ^43^ ^]^ However, its role in HCC remains unclear. Based on the GEPIA database and our cell function experiments, we confirmed that high PSIP1 expression promotes HCC progression and induces resistance to lenvatinib. Furthermore, we observed a significant correlation between PSIP1 expression and disease‐free and OS in patients with HCC, providing further evidence of PSIP1's potential as a downstream target of circRNA‐mTOR. Cai et al. reported that circRNA‐SORE, present in sorafenib‐resistant HCC cells, binds to the oncogenic protein YBX1, preventing its degradation by the E3 ubiquitin ligase PRP19, enhancing sorafenib resistance in HCC.^[^ ^8^ ^]^ These studies suggest that circRNAs alter total amount of RBP by stabilizing proteins in the context of drug resistance in HCC. However, we did not notice any change in PSIP1 expression in response to circRNA‐mTOR application. Another study highlighted M6A‐modified hsa_circ_00 58493, which binds to RBP named YTH Domain‐containing protein 1 and promotes the translocation of circRNA from the nucleus to the cytoplasm, accelerating HCC progression.^[^ ^44^ ^]^ Similarly, CircBACH1 (hsa_circ_00 61395) promotes the translocation of RBP called HuR from the nucleus to the cytoplasm, alleviating its inhibitory effect on p27 translation and facilitating HCC growth.^[^ ^45^ ^]^ Consistent with these two findings, our study shows that circRNA‐mTOR facilitates the translocation of PSIP1 from the cytoplasm to the nucleus. Studies have shown that an increase in nuclear PSIP1 levels leads to reduced ubiquitination and degradation of c‐Myc, a tumor cell stemness marker. These findings are consistent with our previous finding that the circRNA‐mTOR pathway promotes HCC stemness. Our study highlighted the involvement of PSIP1 and circRNA‐mTOR in HCC progression, drug resistance, and stemness, expanding our understanding of the complex mechanisms underlying HCC pathogenesis. Thus, our study provides valuable insights into potential therapeutic strategies that target PSIP1 and circRNA‐mTOR in HCC.

In this study, we analyzed clinical samples and data to investigate the diagnostic value and clinical significance of circRNA‐mTOR. Our in vitro and in vivo experiments further validate these findings and suggest that circRNA‐mTOR holds potential as a biomarker for HCC. Moreover, circRNA‐mTOR has significant therapeutic implications in patients with different HCC stratifications. However, our study had a few limitations. E.g., to strengthen the robustness of our findings, obtaining a larger set of clinical samples, particularly drug‐resistant HCC cells and tissue samples collected before and after lenvatinib treatment, is essential. Additionally, although our research demonstrated that circRNA‐mTOR promotes targeted drug resistance by increasing HCC stemness, other pathways may also be involved. Therefore, further investigations are required to elucidate the specific mechanisms through which circRNA‐mTOR promotes drug resistance. Despite these limitations, our study provides valuable insights into the diagnostic and therapeutic potential of circRNA‐mTOR for HCC treatment. Future studies with larger sample sizes and a comprehensive exploration of the associated pathways will deepen our understanding of the clinical implications of circRNA‐mTOR in HCC management.

In conclusion, our findings suggest that circRNA‐mTOR plays a crucial role in maintaining lenvatinib resistance in HCC. By binding to PSIP1, circRNA‐mTOR affects the nuclear translocation of PSIP1 and promotes HCC progression and lenvatinib resistance via the PSIP1/c‐Myc axis. These results lay an experimental foundation for targeted drug therapies in HCC and offer new perspectives and methods for understanding HCC development. Furthermore, they hold significant importance for identifying new molecular markers and therapeutic targets to reduce drug resistance in HCC.

## 4 Experimental Section

### Tissue Samples

The clinical tissue samples used in this study were obtained from patients who underwent surgery at the Third Affiliated Hospital of Sun Yat‐sen University. A total of 156 pairs of clinical tissue samples collected between December 2012 and September 2018 were included in the study. The follow‐up period extended until August 2021. The study was conducted with the approval of the Ethics Committee of the Third Affiliated Hospital of Sun Yat‐sen University, and informed consent was obtained from all patients. All procedures were carried out in strict adherence to ethical requirements.

### Sphere Formation Assay

The medium formulation for sphere formation assay was shown in the Table S1 (Supporting Information).^[^ ^6^ ^]^ To perform the sphere formation assay, cells to be tested (n = 2 000) were seeded in low‐adhesion 6‐well plates (Corning, USA). The cells were incubated in a constant temperature incubator for 10–14 days, with fresh medium added at 3‐day intervals. To examine the ability of cells to self‐renew after serial passage, a secondary sphere formation assay was required. After the incubation period, the sphere formation was observed, photographed, and counted. The Sphere Volume (SV) was calculated using the formula: SV = 1/2 * major diameter * (the square of the minor diameter). The Sphere Formation Efficiency (SFE) was calculated as the ratio of the number of cell spheres with a diameter ≥ 75 µm to the total number of cells.

### RNA Nucleoplasmic Separation Assay

For the RNA nucleoplasmic separation assay, cell samples were washed twice with sterile PBS and centrifuged at 2 000 rpm for 10 min to collect the cell pellet. The cell pellet was then processed using the Cytoplasmic & Nuclear RNA Purification Kit (Norgen Biotek, Canada) according to the manufacturer's instructions. The purified RNA samples were either used immediately for reverse transcription and qPCR experiments or stored at – 80 °C.

### RNA Pulldown and Liquid Chromatograph‐Mass Spectrometer (LC‐MS)

The Pulldown probe (U‐biotin labeling) of hsa_circ_0 009792 used in this study was designed and synthesized by Guangzhou Bersinbio Co., LTD (China). The Probe sequence was shown in the Table S2 (Supporting Information). Briefly, the main trial procedures included: Formation of RNA secondary structure; Preparation of probe‐magnetic beads; Extraction of whole cell proteins; Removal of nucleic acids and pre‐washing; RNA pulldown; Collection of RNP; Silver staining. RNA pulldown Kit (Bersinbio, China) was used to perform the operations. Next, the pulldown complex and its control were analyzed by enzyme digestion, mass spectrometry detection (Q Exactive, Thermo Scientific, USA) and database retrieval.

### RNA Immunoprecipitation (RIP)

The RNA Immunoprecipitation assay was performed to investigate the interaction between hsa_circ_0 009792 and PSIP1 (Santacruz, USA). The assay involved cell treatment and lysis, removal of DNA, equilibrium of protein A/G magnetic beads, immunoprecipitation, RNA extraction, reverse transcription, and PCR amplification, followed by agarose gel electrophoresis. The RNA Immunoprecipitation Kit (Bersinbio, China) was used for these procedures.

### Protein Nucleoplasmic Separation Assay

For the protein nucleoplasmic separation assay, cell samples were washed twice with sterile PBS, centrifuged at 2 000 rpm for 10 min to collect the cell pellet, and then processed using the Nuclear and Cytoplasmic Protein Extraction Kit (KeyGEN, China) according to the manufacturer's instructions. The purified protein samples were used for concentration determination and Western Blot analysis.

### Co‐Immunoprecipitation (CO‐IP)

CO‐IP experiments were performed to investigate the interaction between PSIP1 (Santacruz, USA) and c‐Myc (Abcam, UK). The assay involved cell treatment and lysis, incubation of proteins with antibodies, preparation of Protein‐A/G magnetic bead suspensions, binding of Protein‐A/G magnetic beads to antibodies, washing and elution of the protein, and Western blot analysis. The Co‐Immunoprecipitation Kit (Bersinbio, China) was used for these procedures.

### Establishment of Subcutaneous Xenograft Model in Nude Mice

The study utilized 4‐week‐old male BALB/c nude mice housed in a specific pathogen‐free (SPF) grade animal facility. The animal experiment was approved by the ethics committee of the Guangdong Weisheng Pharmaceutical Technology Co., Ltd, and all animal procedures followed ethical guidelines. The mice were acclimated to the SPF environment for 1 week before the experiment. Cells with a concentration of 5 × 10^7^ cells/mL, mixed with matrigel, were subcutaneously inoculated into the mice. The subcutaneous tumor formation of mice was regularly observed and recorded every week. For the lenvatinib (Lenvima, Japan) treated mice, lenvatinib (4mg k^−1^g/day) was administered by gavage daily from week 4. According to the purpose of the experiment, the nude mice were sacrificed at the 6th or 8th week, and the subcutaneous tumor tissue was completely removed, photographed, weighed, and recorded. The tumor tissue could be used for subsequent immunohistochemical staining and other experiments. Tumor volume was calculated using the formula: 1/2 * major diameter * the square of the minor diameter.

### Statistical Analysis

Statistical analysis and data visualization were performed using GraphPad Prism, Image J, SPSS software, and R software. The Figdraw platform (https://www.figdraw.com/) was used for pattern diagram creation. Quantitative data were presented as mean ± standard deviation (SD). The following principles were applied for data analysis. Numerical Variables: For normally distributed data with homogeneous variances, T‐test (two groups) or One‐way ANOVA (three groups); For normally distributed data with unequal variances, Welch T‐test (two groups) or Welch One‐way ANOVA (three groups); For non‐normally distributed data, Wilcoxon test (two groups) or Kruskal‐Wallis test (three groups). Categorical Variables: For data with theoretical frequency > 5 and total sample size ≥ 40, Chi‐square test; For data with theoretical frequency 1–5 and total sample size ≥ 40, Yates' correction; For data with theoretical frequency < 1 or total sample size < 40, Fisher's exact test. Survival analysis was depicted using Kaplan‐Meier survival curves, with statistical differences evaluated by the log‐rank test. For the determination of IC50, log‐logistic analysis was used. *P* value < 0.05 was considered as statistically significant.

### Additional Experimental Section

Details were provided in the Supporting Information and Experimental Section.

## Conflict of Interest

The authors declare no conflict of interest.

## Author Contributions

Y.T., F.Y., and M.C. contributed equally to this work. Y.T., M.D., and Z.Y. were involved in the conception and design of the study. Y.T. performed most of the experiments, analyzed data, and wrote the manuscript. F.Y. and M.C. performed some of the experiments and analyzed data. Y.R., Y.L., G.Y., Z.Z., H.L., Z.X., and Z.H. collected the data and performed the follow‐up. N.L., M.D., and Z.Y. reviewed and edited the manuscript. Y.T., M.D., and Z.Y. provided resources and administrative support. All authors reviewed the final version of the manuscript, and agreed with its content and submission.

## Supporting information



Supporting Information

Supplemental Dataset 1

Supplemental Dataset 2

## Data Availability

The dataset used and/or analyzed during the current study are available from the corresponding authors on reasonable request. The data are not publicly available due to privacy
