# Supplementary material for: CircRNA‐mTOR Promotes Hepatocellular Carcinoma Progression and Lenvatinib Resistance Through the PSIP1/c‐Myc Axis
Source: Adv Sci (Weinh). 2025 Apr 15;12(20):2410591. doi: 10.1002/advs.202410591 (PMC12120768; doi:10.1002/advs.202410591)

**Supplementary Materials and Methods**

**Cell lines and cell culture**

Human normal liver cell line L02 and human hepatocellular carcinoma cell lines were purchased from Shanghai Institute of Cell Biology of Chinese Academy of Science (Shanghai, China). All cells were cultured with DMEM medium (Gibco, USA), containing 100 U/ml penicillin (Sigma, USA), 100 μg/ml streptomycin (Sigma, USA), and 10% heat-inactivated foetal bovine serum (FBS) (PAN Biotech, Germany). But the human hepatocellular carcinoma cell lines were cultured with DMEM/F12 medium (Gibco, USA) according to the formula in the sphere formation assay. At the same time, it was necessary to add reagents or drugs such as lenvatinib (Selleck, USA) according to the experimental purpose in different experiments. All cells were grown in a humidified incubator of 5% CO_2_ at 37°C.

**Total RNA extraction and reverse transcription**

Total RNA was extracted from tissue samples or cells using TRIzol (Ambion, USA) method. The cell samples could be directly treated by adding an appropriate amount of TRIzol, but the tissue samples should be ground into powder with adding liquid nitrogen before adding TRIzol. Chloroform (Macklin, China) was then added (volume ratio TRIzol: chloroform = 5:1), left on ice for 5 min, and then centrifuged at maximum speed for 20 min at 4℃. The supernatant was carefully aspirated, an equal volume of Isopropanol (Macklin, China) was added, thoroughly mixed, left on ice for 10 min, and then centrifuged at maximum speed for 10 min at 4℃. At the end of centrifugation, the upper liquid was discarded, pre-cooled absolute ethanol (Macklin, China) was added, centrifuged at the maximum speed for 5 minutes at 4°C, and the operation was repeated once. The supernatant was discarded and DEPC water (ThermoFisher, USA) was added to completely dissolve the precipitate, then the concentration and purity of RNA were determined by using a Nanodrop 2000 spectrophotometer (ThermoFisher, USA), and then the next step experiment or temporarily stored at -80℃. The cDNA was synthesized from circRNA with PrimeScript RT kit (TaKaRa, Japan) and gDNA Eraser kit (TaKaRa, Japan) as required; The cDNA was synthesized from mRNA with HiScipt® III RT SuperMix for qPCR (+gDNA wiper) kit (Vazyme Biotech, China) as required; The cDNA was synthesized from microRNAs (miRNAs) with recombination RNase inhibitor (TaKaRa, Japan), dNTP Mixture (TaKaRa, Japan) and Reverse Transcriptase XL kit (AMV) (TaKaRa, Japan) as required.

**Extraction of genomic DNA (gDNA)**

According to the purpose or need of the experiment, cells were collected and then manipulated under the guidance of the TIANamp Genomic DNA Kit (Tiagen Biocheh, China).

**Real-time quantitative polymerase chain reaction (RT-qPCR)**

RT-qPCR was carried out by using the ChamQ Universal SYBR qPCR Master Mix kit (Vazyme Biotech, China) on the Roche LightCycler 96/480 system (Roche, Switzerland). GAPDH or U6 was used as the internal control as needed. The relative expression level was calculated by the 2^-∆∆Ct^ method. The sequences of specific primers or probe applied in this study were listed in Supplementary Table S2.

**Actinomycin D treatment assay**

The target cells were plated in 6-well plates one day ahead of time. On the second day, 2mL complete medium with actinomycin D (10μg/mL) (Acmec, China) was added to each well, and cell RNA was extracted at 0h, 4h, 8h, and 12h. The expression of hsa_circ_0009792 (circRNA-mTOR) and parent gene mTOR (mRNA-mTOR) were detected at four time points according to the RT-qPCR method described above.

**RNase R resistance assay**

The RNA of the target cells was extracted as described above, and after the RNA concentration was measured, the solution containing 1μg RNA was taken. 7μL DEPC water, 2μL 10× Reaction Buffer, and 3U RNase R (Geneseed, China) reagent were added and incubated in the constant temperature incubator at 37 °C for 20 minutes. After the end of the reaction, the centrifuge tube was placed in a water bath at 70 °C for 10 minutes to inactivate the enzyme. The expression of hsa_circ_0009792 (circRNA-mTOR) and parent gene mTOR (mRNA-mTOR) were detected according to the RT-qPCR method described above.

**Sequencing analysis**

The sequence of hsa_circ_0009792 (data from circBase database) is:

CATCCAGAGATACGCTGTCATCCCTTTATCGACCAACTCGGGCCTCATTGGCTGGGTTCCCCACTGTGACACACTGCACGCCCTCATCCGGGACTACAGGGAGAAGAAGAAGATCCTTCTCAACATCGAGCATCGCATCATGTTGCGGATGGCTCCGGACTATGACCACTTGACTCTGATGCAGAAGGTGGAGGTGTTTGAGCATGCCGTCAATAATACAGCTGGGGACGACCTGGCCAAGCTGCTGTGGCTGAAAAGCCCCAGCTCCGAG

The looping sequence is: CCCCAGCTCCGAGCATCCAGAGATACGC. The PCR products were sequenced and analyzed, and the alignment of the sequences further confirmed the presence of circRNA. PCR products were sequenced using the following primers: forward primer 5'-CGGACTATGACCACTTGACTCTG-3'; reverse primer 5'-CGGATGAGGGCGTGCAGTGT-3'.

**Agarose gel electrophoresis**

An appropriate amount of agarose (Biowest, Spain) was configured into a suitable concentration of TAE (KeyGEN, China) agarose electrophoresis buffer, followed by heating, addition of EB terminator (KeyGEN, China), and gelation. The sample to be tested and loading buffer (KeyGEN, China) were mixed in proportion and added to the wells for electrophoresis. After electrophoresis, ultraviolet exposure imaging was performed in the gel image processor (Tanon, China).

**Fluorescence In Situ Hybridization (FISH)**

The nuclear reference U6 probe (Cy3 labeled) and cytoplasmic reference 18S probe (Cy3 labeled) used in this study were designed and synthesized by Guangzhou Ribobio Co., LTD (China). The FISH probe of hsa_circ_0009792 (labeled with 6-FAM at the 5') was designed and synthesized by Guangzhou Bersinbio Co., LTD (China). The sequence was shown in the Supplementary Table S2. And then manipulated under the guidance of the RiboTM Fluorescent In Situ Hybridization Kit (Ribobio, China). Fluorescence imaging was subsequently performed using fluorescence microscopy (Zeiss, Germany) or confocal microscopy (Leica, Germany).

**Expression vector construction and transfection**

According to the purpose or need of the experiment, the hsa_circ_0009792 cDNA was enlarged and then cloned into the pLO5-ciR expression vector (Geneseed, China), and pLO5 vector was used as the control. The standard of successful vector construction was normal sequencing peak map, no miscellaneous peaks or overlapping bands, and the sequence comparison was consistent. Moreover, we also constructed the lentiviral expression vector (GeneChem, China). Cells were transfected by using Lipofectamine 3000 (Invitrogen, USA) according to the manufacturer's instructions.

**CRISPR/Cas9**

Packaging the lentivirus by using the lentiCRISPR v2 vector inserted with PSIP1-sgRNA (Beyotime) (1500ng) , the packaging plasmid psPAX2 (Addgene), and the envelope plasmid VSV-G (Addgene). Then, virus infection, monoclonal screening, and detection were carried out according to the above experimental methods.

**Interference system construction and transfection**

The ASO interference system and the shRNA interference system used in this study were designed and synthesized by Guangzhou Ribobio Co., LTD (China). All the target sequences of the interference systems were shown in the Supplementary Table S2. Cells were transfected by using Lipofectamine 3000 (Invitrogen, USA) according to the manufacturer's instructions.

**Cell Counting Kit 8 (CCK8) cell proliferation assay**

Equal numbers of cells to be tested (n=1,000-3,000) were plated in 96-well plates and then cultured for 1day, 2day, 3day, 4day, 5day or 6day in the constant temperature cell incubator. The proliferation ability of the cells was measured using the Cell Counting Kit-8 (Rarbio, China) according to the manufacturer's protocol. Absorbance (optical density, OD) was measured at 450nm by using Microplate Reader (Biotek, USA).

**Colony formation assay**

Equal numbers of cells to be tested (n=500-2,000) were plated in 96-well plates until visible colonies appeared. Then, cell colonies were fixed in ice-cold methanol (Macklin, China) for 30 min, stained with 0.1% crystal violet (Sigma, USA) for 30 min, and quantified using Image J software.

**Transwell invasion assay**

Matrigel (Solarbio, China) was added to the top chamber of the transwell filter (Corning, USA) on the first day. The next day, cells to be tested were seeded into the upper chamber with 100μl of serum-free medium, and the complete medium was added to the lower chamber. Then, it was cultured for 24h in the constant temperature cell incubator. Next, the cells in the upper chamber were removed, and the invaded cells were fixed in ice-cold methanol (Macklin, China) for 30 min, stained with 0.1% crystal violet (Sigma, USA) for 30 min. The number of cells that penetrated was counted.

**Wound healing assay**

Cells to be tested were plated in 6-well plates until the cells covered the bottom of the plate. Then, a straight line of uniform width was drawn at the bottom of the plate, and photographs (0h) were taken under a light microscope (Leica, Germany). Then, it was cultured for 48h in the constant temperature cell incubator, and photographs (48h) were taken under a light microscope.

**Detect apoptosis by flow cytometry**

According to the purpose or need of the experiment, the cell samples to be tested were washed twice with sterile PBS, centrifuged at 2000 rpm for 5 min to precipitate. And then manipulated under the guidance of the Annexin V-FITC/PI Apoptosis Detection Kit (Vazyme Biotech, China). Flow cytometry (BD-LSR II, USA) was used to detect and FlowJo software (10.0.7, USA) was used to analyze the results.

**The Half Maximal Inhibitory Concentration (IC50)**

Equal numbers of cells to be tested were plated in 96-well plates on the first day. The next day, 100μl lenvatinib (Selleck, USA) complete medium with different concentration gradients (0μM, 0.625μM, 1.25μM, 2.5μM, 5μM, 10μM, 20μM, 40μM) was added to each well, and at least 3 multiple wells were set up for each concentration. At the same time, the blank control group was set up and put back into the constant temperature incubator for 48h. After 48h, the OD value (450nm) of each well was determined using the CCK8 method. IC50 values were calculated from simulated cell survival curves calculated at different concentrations.

**Protein extraction, concentration determination and Western Blot**

The protein was extracted from cells or tissues with Protein Extraction Kit (LIFE-Ilab, China), phenylmethylsulfonyl fluoride (LIFE-Ilab, China), and phosphatase inhibitors (LIFE-Ilab, China). Protein concentration was determined with the BCA Protein Quantification Kit (Dingguocs, China). Equal protein samples were resolved by sodium dodecyl sulfate–polyacrylamide gel electrophoresis (SDS-PAGE) (Resolving Gel Buffer, Bio-Rad, USA; Stacking Gel Buffer, Bio-Rad, USA; 30%Acr-Bis, biosharp, China; Tris Base, solarbio, China; SDS, Biofroxx, China; Ammonium Persulfate, Macklin, China; TEMED, BOSTER, China) and transferred to polyvinylidene fluoride membranes (Merck millipore, Germany). After blocking (Skim milk powder, Biofroxx, China), membranes were immunoblotted overnight at 4 °C with antibody solution. The antibodies used in this study: PSIP1, Santacruz, USA; mTOR, Abcam, UK; p62, Abclonal, China; LC3B, Abclonal, China; c-Myc, Abcam, UK; Nanog, CST, USA; Oct4, CST, USA; SOX2, CST, USA; EGFR, Affinity Biosciences, China; β-actin, Abclonal, China; GAPDH, Affinity Biosciences, China; Histone H3, Abcam, UK. After incubation with the secondary antibody (Abclonal, China), ECL imaging (Advansta, USA) was performed on an automatic chemiluminescence image analysis system (Tanon, China).

**Ubiquitination detection**

Construct the required plasmid, transfect the cells with the plasmid for 20 hours, add MG132 (Selleck) at a final concentration of 10 μM for 6 hours, collect and lyse the cells. Incubate the sample with anti HA antibodies and protein A-Sepharose (Sigma-Aldrich), separate by SDS-PAGE and analyze by Western blotting.

**Fluorescence in situ hybridization and immunofluorescence colocalization**

In this study, the assay was used to explore the co-localization of hsa_circ_0009792 and related proteins including PSIP1 (Santacruz, USA) and c-Myc (Abcam, UK). Briefly, the main trial procedures included: The slides of cells; Immobilization and permeabilization of cells; Incubation of the probe; Washing and Immunofluorescence co-localization; DAPI staining; Antifade sealed section and observation by confocal microscopy (Leica, Germany). The RiboTM Fluorescent In Situ Hybridization Kit (Ribobio, China) was used to perform the operations.

**Immunohistochemical (IHC) staining**

Briefly, the main trial procedures included: Tissue paraffin sections were baked at 60°C for 2 hours, and then placed in xylene (Guangzhou Chemical, China) for 15 minutes while they were hot for dewaxing. Different concentration gradients of alcohol (anhydrous alcohol, 95%, 80%, 75%) (Macklin, China) were used for hydration treatment, and EDTA antigen retrieval solution (PH=8, ZSGB-BIO, China) was used in the pressure cooker for 25 minutes. After cooling in running water, it was treated with 3% hydrogen peroxide (Hengjian, China) for 10 minutes to remove endogenous catalase. Soaked with freshly prepared PBS for 3 times, each time for 5 minutes. Added the primary antibody (Ki67, 1:200, Abclonal, China) and incubated overnight at 4℃ in a humidified box. The next day, washed away the primary antibody with PBS, add the secondary antibody (Dako A solution, Denmark), and incubated in a 37°C incubator for 40 minutes. After washing off the secondary antibody with PBS, it was developed with DAB (Dako REAL™, Denmark), and then the nucleus was stained with hematoxylin (Heagene, China).

**Supplementary Tables**

**Table S1**. The medium formulation for sphere formation assay

| **Component** | **Concentration or content** |
| --- | --- |
| DMEM/F12 medium (Gibco, USA) |  |
| Penicillin (Sigma, USA) | 100U/ml |
| Streptomycin (Sigma, USA) | 0.1mg/ml |
| Human recombinant epidermal growth factor (hEGF) (PeproTech, USA) | 20ng/ml |
| Human recombinant fibroblast growth factor (hFGF) (PeproTech, USA) | 20ng/ml |
| Non-essential amino acids (KeyGEN, China) | 1% |
| Glutamine (Gibco, USA) | 1% |
| B27 (Gibco, USA) | 2% |
| Methylcellulose (Yuanye Bio, China) | 1% |

**Table S2**. The sequence list

| Name | Item | Sequences（5'--->3'） |
| --- | --- | --- |
| GAPDH | Forward | GGAGCGAGATCCCTCCAAAAT |
|  | Reverse | GGCTGTTGTCATACTTCTCATGG |
| hsa_circ_0009792  (circRNA-mTOR) | Forward | CGGACTATGACCACTTGACT |
|  | Reverse | TATCTCTGGATGCTCGGAGC |
| hsa_circ_0003110 | Forward | AGAACCGTCTCCGTTCTTCC |
|  | Reverse | TCTCTGGATCCTGCTCCATCA |
| hsa_circ_0064288 | Forward | GCACTGCTCTTGAAAACCCTG |
|  | Reverse | AGACTCGAGTGTGTTGGTGTT |
| hsa_circ_0007061 | Forward | GCTGCCTGAGTACTTGCCTTA |
|  | Reverse | TCCTGCTCCATCATAACGGG |
| hsa_circ_0009792  (Divergent Primer) | Forward | CGGACTATGACCACTTGACT |
|  | Reverse | TATCTCTGGATGCTCGGAGC |
| hsa_circ_0009792  (Convergent Primer) | Forward | CAGAGATACGCTGTCATCCCT |
|  | Reverse | CAAACACCTCCACCTTCTGC |
| GAPDH  (Divergent Primer) | Forward | GGGAGCCAAAAGGGTCATCA |
|  | Reverse | TTGGAGGGATCTCGCCTGA |
| GAPDH  (Convergent Primer) | Forward | GGAGCGAGATCCCTCCAAAAT |
|  | Reverse | GGCTGTTGTCATACTTCTCATGG |
| mTOR | Forward | CAGAGATACGCTGTCATCCCT |
|  | Reverse | CAAACACCTCCACCTTCTGC |
| U6 | Forward | TATCTCTGGATGCTCGGAGC |
|  | Reverse | CTCGCTTCGGCAGCACA |
| PSIP1 | Forward | CGCCAAGATGAAAGGTTATCCC |
|  | Reverse | TTGTGGGTGGCTTTACAGCTC |
| hsa_circ_0009792  (FISH probe) | Sequence | CGTATCTCTGGATGCTCGGAGCTGGGGC |
| hsa_circ_0009792  (ASO-1) | Target Sequences | CAGCTCCGAGCATCCAGAGA |
| hsa_circ_0009792  (ASO-2) | Target Sequences | CTCCGAGCATCCAGAGATAC |
| PSIP1  (shRNA) | Target Sequences | TTTAGGACCAAAGGATATATT |
| hsa_circ_0009792  (Pulldown probe) | Sequence | CATCCAGAGATACGCTGTCATCCCTTTATCGACCAACTCGGGCCTCATTGGCTGGGTTCCCCACTGTGACACACTGCACGCCCTCATCCGGGACTACAGGGAGAAGAAGAAGATCCTTCTCAACATCGAGCATCGCATCATGTTGCGGATGGCTCCGGACTATGACCACTTGACTCTGATGCAGAAGGTGGAGGTGTTTGAGCATGCCGTCAATAATACAGCTGGGGACGACCTGGCCAAGCTGCTGTGGCTGAAAAGCCCCAGCTCCGAGCATCCAGAGATACGCTGTCATCCCTTTATCGACCAACTCG |

**Supplementary Figures**

**Figure S1:** CircRNA-mTOR promotes HCC progression. **A:** CCK8 cell proliferation assay; **B:** Clone formation assay; **C:** Transwell invasion assay, Scale bar, 50 µm; **D:** Wound Healing assay; **E:** Flow cytometry was used to detect apoptosis; **F:** Subcutaneous tumor formation of nude mice in the control (sh-NC) group and the interference hsa_circ_0009792 expression (sh-circ) group; Physical image of the tumor (left); Comparison of tumor growth curve and weight between the two groups (middle); Immunohistochemical staining results of Ki67 expression levels of tumors in the two groups (right) , Scale bar, 50 µm. **P*<0.05; ***P*<0.01; ****P*<0.001.


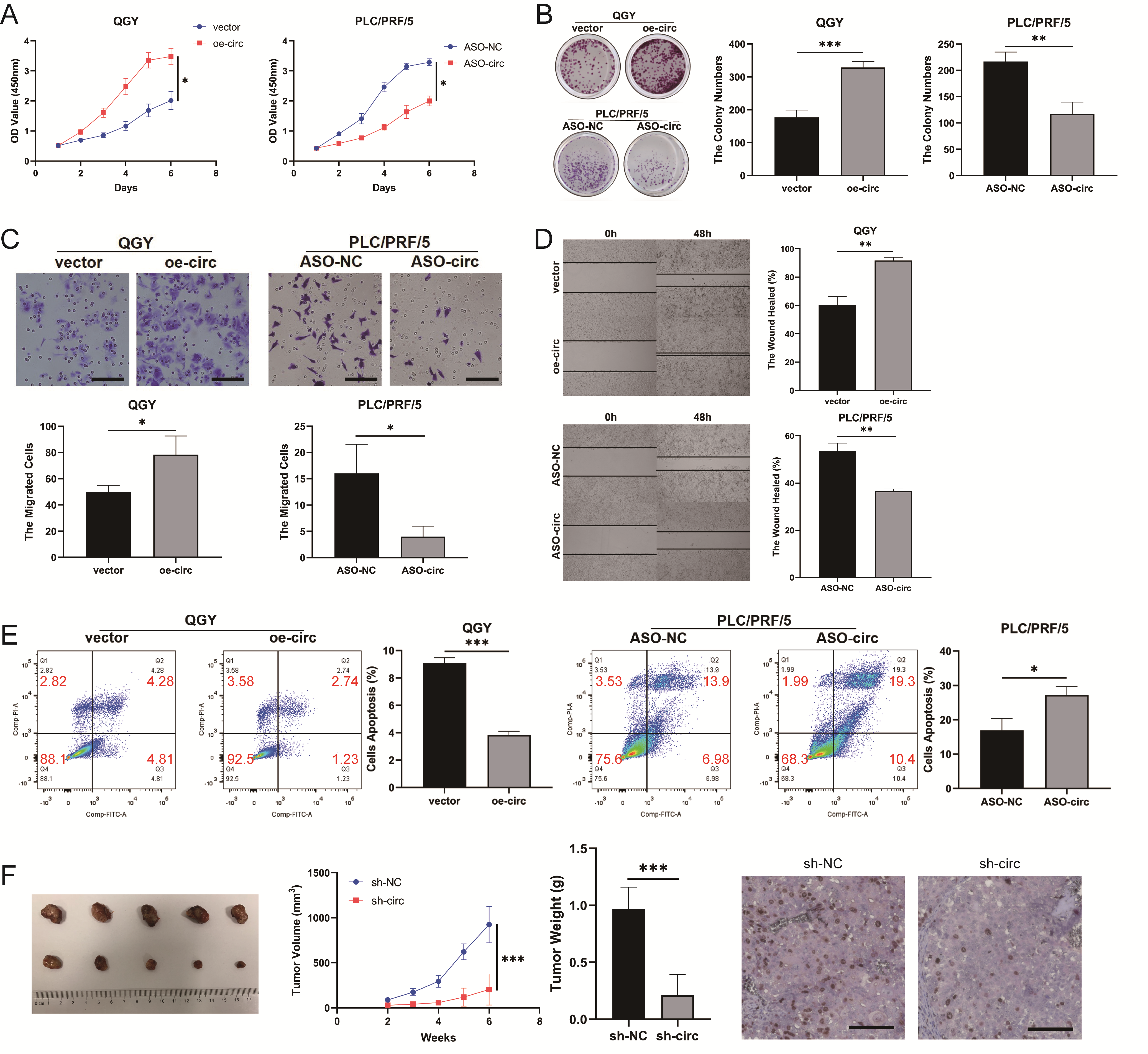


**Figure S2:** CircRNA-mTOR promotes Lenvatinib resistance and the improvement of tumor stemness in HCC. **A:** CircRNA-mTOR significantly increased the lenvatinib IC50 value of HCC cells. The IC50 value for QGY: blank 4.691 (4.26-5.122) μM, vector 4.272 (3.887-4.656) μM, OE-circRNA 8.339 (7.667-9.012) μM; The IC50 value for PLC/PRF/5: blank 4.631 (4.48-4.781) μM, ASO-NC 4.406 (4.261-4.551) μM, ASO-circRNA 2.613 (2.534-2.692) μM. **B:** CircRNA-mTOR promoted the proliferation ability of HCC cells when Lenvatinib was treated; **C:** CircRNA-mTOR promoted the colony formation ability of HCC cells when Lenvatinib was treated; **D:** CircRNA-mTOR down-regulated the apoptosis level of HCC cells when Lenvatinib was treated; **E:** CircRNA-mTOR promoted the stemness level of HCC cells when Lenvatinib was treated, Scale bar, 100 µm. **P*<0.05; ***P*<0.01; ****P*<0.001.


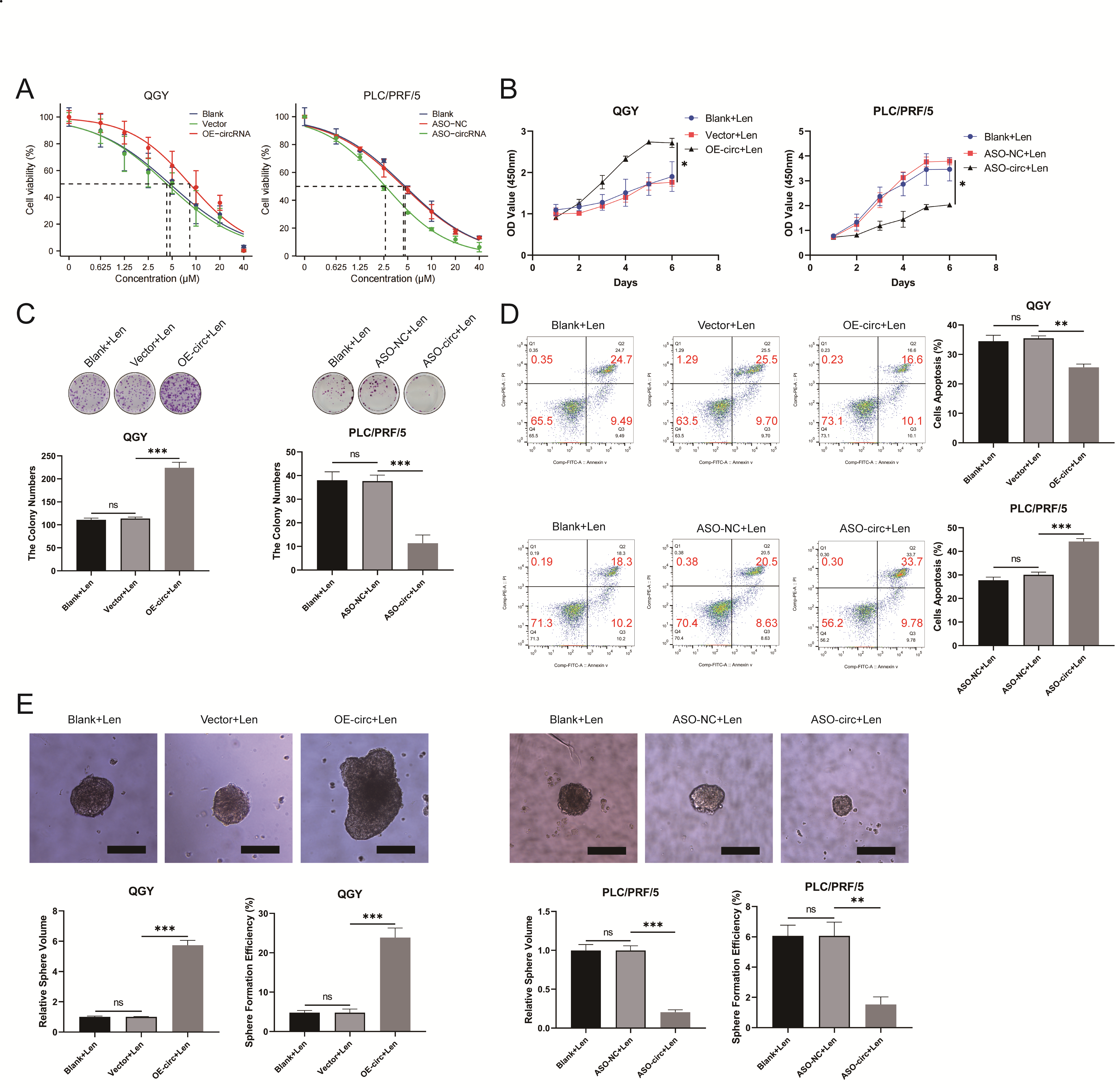


**Figure S3:** Investigation of the downstream mechanism of circRNA-mTOR in HCC. **A:** CircRNA-mTOR did not affect the expression of its parent gene mTOR at the mRNA level; **B:** CircRNA-mTOR did not affect the expression of its parent gene mTOR protein and cell autophagy; **C:** Molecular function results of differential proteins from pulldown and LC-MS experiment after GO analysis; **D:** Biological process results of differential proteins from pulldown and LC-MS experiment after GO analysis; **E:** Cell components results of differential proteins from pulldown and LC-MS experiment after GO analysis; **F:** Analysis results of differential proteins from pulldown and LC-MS experiment in protein classification. ns: no significance. ns: no significance.


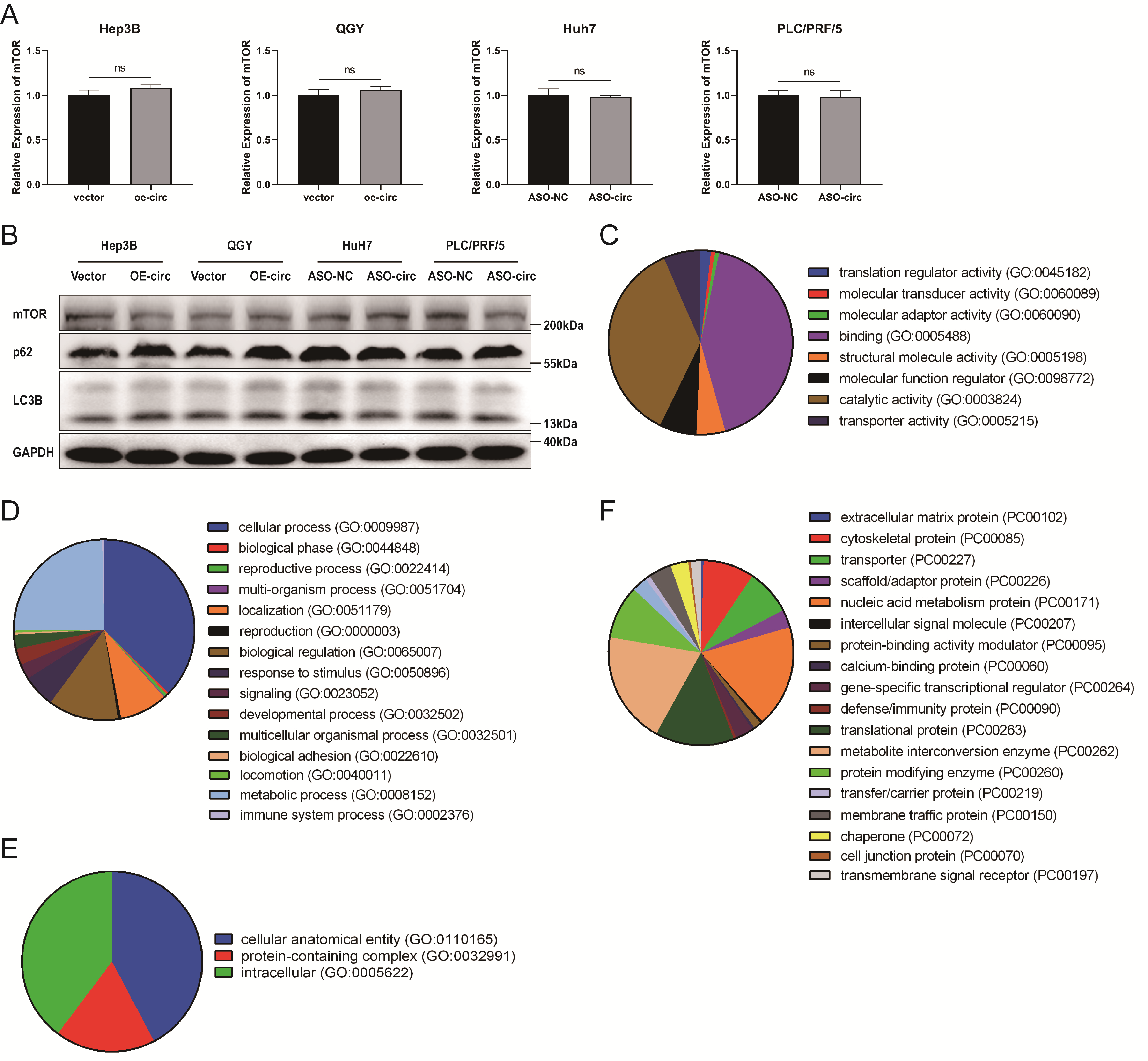


**Figure S4:** PSIP1 promotes the progression of HCC and Lenvatinib resistance. **A:** Related information of PSIP1 in the GEPIA database, expression situation (left), DFS (middle), OS (right); **B:** Interference efficiency of PSIP1; **C:** CCK8 cell proliferation assay showed that PSIP1 promoted the proliferation of HCC cells; **D:** The clone formation assay showed that PSIP1 promoted the colony formation of HCC cells; **E:** Transwell invasion assay showed that PSIP1 promoted the invasion of HCC cells, Scale bar, 50 µm; **F:** Wound healing assay showed that PSIP1 promoted the migration of HCC cells; **G:** Sphere formation experiment showed that PSIP1 promoted the increase of cell stemness in HCC, Scale bar, 100 µm; **H:** PSIP1 promoted Lenvatinib resistance in HCC cells. **P*<0.05; ***P*<0.01; ****P*<0.001.


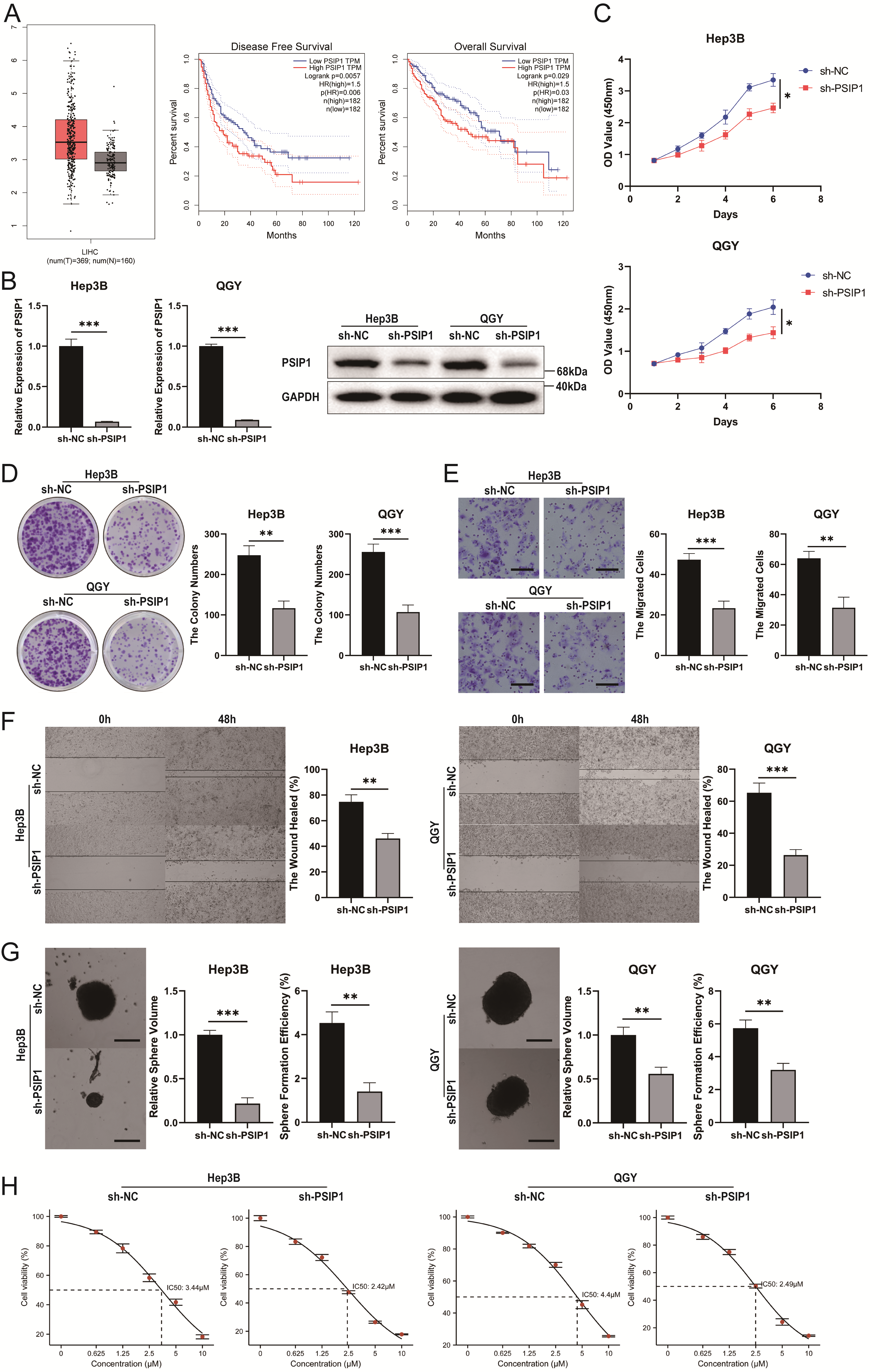


**Figure S5:** CircRNA-mTOR promotes the progression of HCC and Lenvatinib resistance by binding to PSIP1 and affecting its nuclear translocation. **A-G:** After interfering with PSIP1 expression, the promotion and improvement of circRNA-mTOR on the proliferation ability, colony formation ability, invasion and metastasis ability, tumor stemness and IC50 value of Lenvatinib of HCC cells could be partially reversed, and the inhibition of circRNA-mTOR on the apoptosis of HCC cells could be partially reversed; **A:** CCK8 cell proliferation assay; **B:** Clone formation assay; **C:** Transwell invasion assay, Scale bar, 50 µm; **D:** Wound Healing assay; **E:** Flow cytometry was used to detect apoptosis; **F:** Sphere formation experiment, Scale bar, 100 µm; **G:** Lenvatinib IC50; **H:** CircRNA-mTOR did not affect the expression of PSIP1; **I:** The proportion of circRNA-mTOR in the nucleus increased after overexpression; **J:** The proportion of PSIP1 in the nucleus increased after the overexpression of circRNA-mTOR. **K:** The detection of PSIP1 knockout efficiency by RT-qPCR (left) and Western Blot (right). **P*<0.05; ***P*<0.01; ****P*<0.001; ns: no significance.


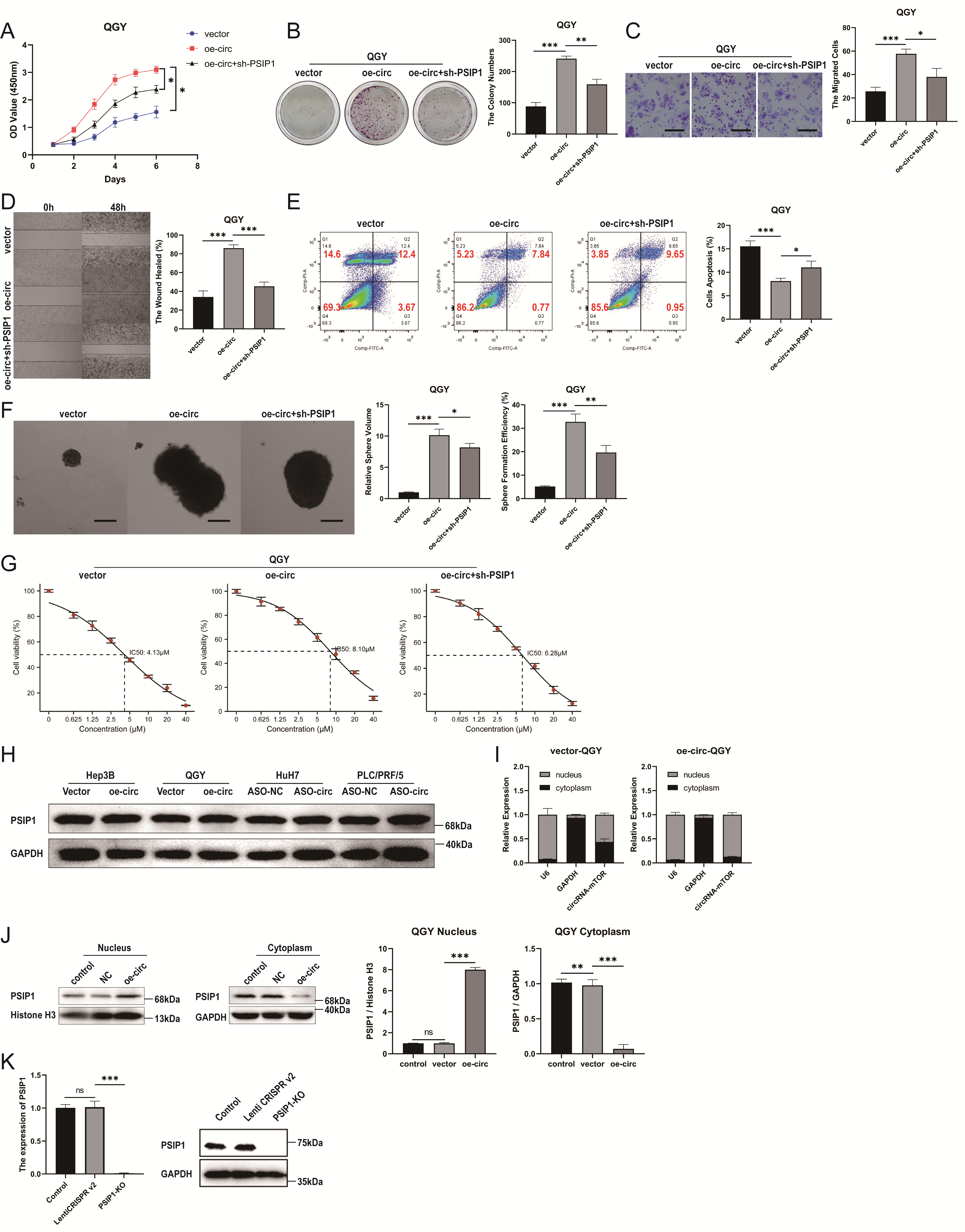

Supplement: Supplementary file 1 — Supporting Information [file ADVS-12-2410591-s002.doc]
